# The effects of mobile health on emergency care in low- and middle-income countries: A systematic review and narrative synthesis

**DOI:** 10.7189/jogh.11.04023

**Published:** 2021-04-03

**Authors:** W Tyler Winders, Stephanie C Garbern, Corey B Bills, Pryanka Relan, Megan L Schultz, Indi Trehan, Sean M Kivlehan, Torben K Becker, Ruth McQuillan

**Affiliations:** 1 School of Public Health, University of Edinburgh, Edinburgh, UK; 2 Department of Emergency Medicine, Alpert Medical School of Brown University, Providence, Rhode Island, USA; 3 Department of Emergency Medicine, University of Colorado School of Medicine, Denver, Colorado, USA; 4 Department of Emergency Medicine, Emory Healthcare Network, Atlanta, Georgia, USA; 5 Department of Pediatrics, Medical College of Wisconsin, Milwaukee, Wisconsin, USA; 6 Departments of Pediatrics and Global Health, University of Washington, Seattle, Washington, USA; 7 Department of Emergency Medicine, Brigham and Women's Hospital, Boston, Massachusetts, and Harvard Humanitarian Initiative, Cambridge, Massachusetts, USA; 8 Department of Emergency Medicine, University of Florida, Gainesville, Florida, USA; 9 Usher Institute of Population Health Sciences and Informatics, University of Edinburgh, Edinburgh, UK

## Abstract

**Background:**

In resource-constrained settings, mobile health (mHealth) has varied applications. While there is strong evidence for its use in chronic disease management, the applications of mHealth for management of acute illness in low- and middle-income countries (LMICs) are not as well described. This review systematically explores current available evidence on the effectiveness of mHealth interventions at improving health outcomes in emergency care settings in LMICs.

**Methods:**

A systematic search of the literature was performed in accordance with PRISMA guidelines, utilizing seven electronic databases and manual searches to identify peer-reviewed literature containing each of three search elements: mHealth, emergency care (EC), and LMICs. Articles quality was assessed using the Grading of Recommendations Assessment, Development and Evaluation (GRADE) criteria.

**Results:**

After removing duplicates, 6498 studies met initial search criteria; 108 were eligible for full text review and 46 met criteria for inclusion. Thirty-six pertained to routine emergency care, and 10 involved complex humanitarian emergencies. Based on the GRADE criteria, 15 studies were rated as “Very Low” quality, 24 as “Low” quality, 6 as “Moderate” quality, and 1 as “High” quality. Eight studied data collection, 9 studied decision support, 15 studied direct patient care, and 14 studied health training. All 46 studies reported positive impacts of mHealth on EC in LMICs.

**Conclusions:**

Mobile health interventions can be effective in improving provider-focused and patient-centered outcomes in both routine and complex EC settings. Future investigations focusing on patient-centered outcomes are needed to further validate these findings.

Mobile phones have evolved from simple communication devices to smartphone platforms with advanced data storage, internet access, and photographic capabilities. These emerging technologies have been widely adopted in health care and fall under the inclusive term mobile health (mHealth), defined by the World Health Organization (WHO) as “medical and public health practice supported by mobile devices, such as mobile phones, patient monitoring devices, personal digital assistants, and other wireless devices.” [1]. Mobile health advances have enabled cutting edge health care in traditionally resource-constrained environments [2].

Mobile health applications improve upon the disproportionately poor health outcomes that low- and- middle-income countries (LMICs) are known to endure [2]. The evidence base for mHealth in LMICs is robust but largely focused on chronic care. There are numerous systematic reviews and meta-analyses detailing the effectiveness of mHealth on the care for various chronic diseases in LMICs, revealing improved medication adherence, symptom control, and lowered risk of death and hospitalization [3-5]. However, mortality associated with routine emergency care (EC) in LMICs is several-fold higher than in high-income countries (HICs) [6,7], and complex humanitarian emergencies (CHEs) are known to exhibit extremely high mortality rates, particularly when they occur in an LMIC [8]. These well documented inequities lend themselves to innovative interventions, such as mHealth. There is a growing body of literature on the use of mHealth for addressing acute care needs in LMICs that has yet to be synthesized. Initial studies on the utility of mHealth in EC in LMICs are promising. The objective of this systematic review is to provide the first formal synthesis of the effects of mHealth as it relates to emergency care in LMICs.

## METHODS

### Search strategies

This systematic review was developed and conducted in collaboration with the Global Emergency Medicine Literature Review (GEMLR) group and the Usher Institute of Population Health Sciences and Informatics (University of Edinburgh). The Usher Research Ethics Group approved systematic review protocol, which was then registered with PROSPERO (registration number CRD42019151080) on December 20, 2019. The published study protocol is available in Appendix S1 of the **Online Supplementary Document** and searchable on PROSPERO. A rigorous search strategy was designed in collaboration with a health sciences medical librarian with the goal of identifying all randomized controlled trials and observational studies that described the effectiveness of mHealth interventions on EC in LMICs. No patients were asked for input in the creation of this article.

Five unique electronic databases were systematically searched: Pubmed, OVID: Global Health (CABI), Embase, Web of Science, and Global Index Medicus. These databases were selected with the goal of including all published literature worldwide. Eligible studies included all randomized controlled trials (RCTs) and observational studies with controls that included each of three major search themes: mobile health, emergency care, and LMIC (see Appendix S2 in the **Online Supplementary Document**). The initial search strategy was developed within Pubmed and adapted for the remaining four databases. Free text terms and standardized MeSH headings/subheadings in the context of Boolean operators and appropriate search term truncation were utilized to optimize sensitivity for relevant literature while minimizing excess search results. The search strategy was optimized via multiple trial searches, verifying that all previously identified relevant studies were included. The reference lists of prior similar reviews and studies likely to meet inclusion criteria were searched manually to both verify search sensitivity and identify other potentially relevant studies that were not identified by the electronic search. A manual grey literature search was also performed via advanced Google searches targeting organizations (eg, World Health Organization and International Committee of the Red Cross) known to publish global EC literature. See Appendix S2 in the **Online Supplementary Document** for the specific search strategies utilized for each database.

### Data processing

After removal of duplicate articles, two independent reviewers screened each title and abstract. Covidence, an online systematic review platform, was utilized to facilitate study screening [9]. The primary author (WTW) screened all titles and abstracts, and second reviewers (CB, SG, PR, MS) each screened one quarter. Discrepancies were resolved by a third reviewer from the GEMLR group. The same procedure was followed for full-text screening. Articles were excluded if they were not in English or Spanish, clearly irrelevant to the topic (such those with a focus on chronic disease management and/or care in high-income countries), not emergency care, considered social media (internet based applications, such as WhatsApp or Twitter, that allow exchange of user created content [10]), not mHealth, evaluating non-health care related outcomes (such as economic/monetary outcomes, supply chain efficiency, management or process outcomes), not undertaken in a LMIC, or abstract-only publications.

Data extracted from final manuscripts included: author, publication date, location, study type, mHealth intervention type, setting (routine EC or CHE), methods, primary outcomes, secondary outcomes, and limitations. Study quality was assessed using the Grading of Recommendations Assessment, Development, and Evaluation (GRADE) criteria [11]. Criteria proposed by the Preferred Reporting Items for Systematic Reviews and Meta-analyses (PRISMA) statement were adhered to in reporting [12].

### Data analysis

The study aims resulted in significant heterogeneity in study type, methodology, intervention, and outcomes. Even when studies were stratified into four categories of mHealth interventions (decision support, data collection, direct patient care, and health education), significant heterogeneity remained. The requisite criteria for formal meta-analyses and funnel plots were not met, and therefore, a qualitative analysis and narrative synthesis was undertaken. Thematic analysis was undertaken focusing on the effectiveness of the health outcomes in each of the four different categories of mHealth applications in EC in LMICs.

## RESULTS

**Figure 1:**
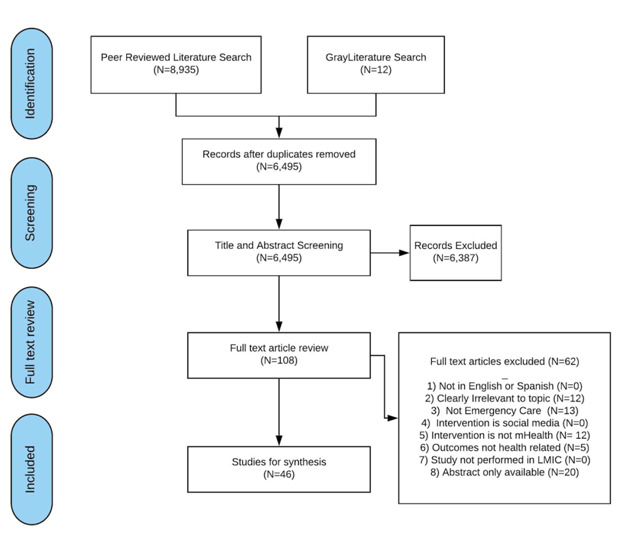
PRISMA flow diagram.

A total of 8947 articles were identified for screening via database searches, and 12 were included based on the grey literature search (**Figure 1**). After removal of duplicates, 6495 unique articles were included in title and abstract screening. A total of 108 articles met criteria for full text screening. 62 articles were excluded during full text screening, leaving 46 articles that met criteria for inclusion.

Of the 46 included studies, 45 were published in peer-reviewed journals, and one was a grey literature publication. Thirty-six encompassed routine EC, and 10 involved CHEs. Geographically, publications originating from all six WHO Member State Regions were included [13]. Based on the World Bank development indices, this systematic review encompassed 16 studies from low-income countries, 12 studies from lower-middle income countries, and 17 studies from upper-middle income countries. A single study reported data from three distinct countries, World Bank development classifications, and regions [14]. **Table 1** provides general study characteristics.

**Table 1:** Study characteristics

WHO Regional Office*	N (%)
AFRO	19 (42.2%)
AMRO	7 (15.5%)
SEARO	10 (22.2%)
EURO	2 (4.4%)
EMRO	4 (8.8%)
WPRO	3 (6.6%)
World Bank Development Index*		
Low income	16 (35.5%)
Lower-middle income	12 (26.6%)
Upper-middle income	17 (37.7%)
**Setting**	
Complex humanitarian crisis	10 (21.8%)
Routine emergency care	36 (78.2%)
**Methodology**	
RCT	7 (15.2%)
Observational cohort	21 (45.7%)
Observational pre/post	18 (39.1%)
**mHealth intervention**	
Data collection	8 (17.4%)
Decision support	9 (19.6%)
Direct patient care	15 (32.6%)
Health trainings	14 (30.4%)
**GRADE quality**	
High	1 (2.2%)
Moderate	6 (13.0%)
Low	24 (52.2%)
Very low	15 (32.6%)

WHO – World Health Organization, AFRO – African Regional office, AMRO – American regional office, SEARO – South-East Asian regional office, EURO – European regional office, EMRO – Easter Mediterranean regional office, WPRO – Western Pacific regional office, RCT – randomized controlled trial

*Finette et al. reported data from Burkina Faso (AFRO/low income), Ecuador (PAHO/upper-middle income), and Bangladesh (SEARO/lower-middle income) [14].

Methodologically, 7 studies were randomized controlled trials (RCTs), 21 were observational cohorts with control groups, and 18 were observational pre/post studies. Of the four prespecified categories of mHealth interventions delineated in this systematic review, 8 studied data collection interventions [15-22], 9 studied decision support interventions [14,23-30], 15 studied direct patient care interventions [31-45], and 14 studied health training interventions [46-59]. **Table 2** provides detailed study characteristics. The four prespecified subtypes of mHealth interventions were utilized to structure the thematic analysis.

**Table 2:** Detailed study descriptions, sorted by primary mHealth intervention

Author (year), Country, World Bank classification	Study type	Intervention	Participants	Effect	GRADE score
**Data collection**					
Bengtsson (2011), Haiti, Low income [16]	Observational cohort	Sim card data from Haiti's largest provider was used to track population movements during the 2010 Haiti earthquake and cholera outbreak in order to (1) estimate the magnitude and trends of population, (2) compare the results with other data sources, and (3) to assess the feasibility of using mobile phone network data to rapidly track population movements during humanitarian crises	1.9 million SIM cards that made at least one call both pre-earthquake and during the last month of study were included in geospatial analyses	Estimated net outflow of people (outflow minus inflow) corresponded to 20% of the Port-au-Prince (PaP) pre-earthquake population.	Very low
				Sim-card based estimates of the geographical distributions across Haiti were similar to the estimates derived from the retrospective UNFPA study, which included a representative sample of 2500 households in PaP and among which 2921 persons had left PaP following the earthquake	
Bengtsson (2015), Haiti, Low income [15]	Observational cohort	Anonymous mobile phone data was used to model predictability of cholera outbreak in geographic regions of Haiti the 2010 cholera outbreak in 78 regions of Haiti. A prospective model was created based on population mobility that predicts infectious pressure and was compared to retrospective data over a period of two months. For each newly infected area, the infectious pressure was correlated with the average daily number of cases.	Movements of 2.9 million anonymous mobile phone SIM cards were used to create a national mobility network	Risk of an area experiencing an outbreak within seven days showed strong dose-response relationship with the mobile phone-based infectious pressure estimates. The mobile phone-based model performed better (AUC 0.79) than the retrospectively optimized gravity models (AUC 0.66 and 0.74, respectively). Infectious pressure at outbreak onset was significantly correlated with reported cholera cases during the first ten days of the epidemic (*P* > 0.05)	Low
Davies (2019), South Africa, Upper-middle income [17]	Observational cohort	An mHealth tool, MalariaConnect, was compared with traditional paper-based reporting methods in health facilities in South Africa during an outbreak in 2017. Generated data for both were compared for timeliness, completeness, and precision.	39 facilities reported a total of 1047 malaria cases via paper methods and 1000 via MalariaConnect during the study period.	Timeliness of reporting increased significantly using MalariaConnect with 0.63 days to notification compared to 5.65 days using the paper system (*P* < 0.05). The completeness of reporting was significantly higher for the paper system (100% completion; *P* < 0.05). There was a moderate association between data precision and the reporting system (*P* < 0.05)	Low
El-Khatib (2018), Central African Republic, Low income [18]	Observation pre/post + external control	To evaluate the effectiveness of an Android-based disease surveillance app, Argus, as compared to paper -based surveillance, a 15-week pilot project was undertaken in 21 health centers in the MK district (MK2016). Results were compared to the usual paper-based surveillance in MK the year prior (MK 2015) and simultaneously in an adjacent health district, NM (NM 2016).	A total of 3403 cases and 63 deaths were notified in the Weekly Reports during the 15-week period.	Overall, the median completeness of Weekly Reports was significantly higher in MK 2016 (81%, IQR 81-86%) than in MK 2015 (29%, IQR 24-36%) and NM. 2016 (52%, IQR 48-57%) (*P* < 0.01). Similarly, the median timeliness of complete reports was significantly higher in MK 2016 (50%, IQR 39-57%), than in MK 2015 (19%, IQR 19-24%) and NM 2016 (29%, IQR 24-36%) (*P* < 0.001).	Moderate
Feng (2018), Liberia, Low income [19]	Observational cohort	The aim was to evaluate the efficacy SMS based surveys to collect individual-level health data in resource strained settings. A national SMS-poll and collected data about individual-level health and health-seeking behavior during the Ebola outbreak from 6694 individuals from March to June 2015 in Liberia. Authors compared their findings to a recent national household survey.	5900 individuals were compared to 5337 propensity score matched control subjects.	The matched subgroups had similar patterns of delivery location in aggregate. Additionally, facility-based deliveries were significantly decreased during, compared to after the outbreak (*P* < 0.05) consistent with findings from retrospective studies using health care-based data.	Low
Jia (2015), Sierra Leone, Low income [20]	Observational cohort	The aim was to evaluate the effectiveness of using cell phone message for Ebola hemorrhagic fever (EHF) syndromic surveillance in a high risked community in Sierra Leone. A longitudinal data analysis of the monthly cumulative confirmed EHF cases and mortalities collected by both the traditional sentinel and community cell phone syndromic surveillance from August to October 2014 was undertaken.	A total of 129 and 49 EHF suspect and confirmed cases respectively were recorded.	There was a strong positive linear correlation (R = 0.8493) between cell phone syndromic surveillance reported EHF suspect cases and deaths when data was stratified for chiefdoms in Moyamba District. There was weak correlation between the traditional sentinel surveillance data for suspect cases and deaths (R = 0.0013) when data was stratified for chiefdoms in Moyamba District for August-September 2014 and correlation was also weak (R = 0.00024) for EHF suspect cases and seropositivity.	Very low
Toda (2016), Kenya, Lower-middle income [21]	RCT	This RCT study aimed to assess mSOS (a formatted text-messaging system that enables communication and monitoring between health care facility workers and Ministry of Health managers) vs routine paper-based protocol for communicable disease reporting. Health facilities were randomized to routine procedure or to the mSOS intervention, which included formal training on the use of mSOS.	135 health facilities randomized to 67 intervention and 68 control	In the per-protocol analysis, the percentage of cases for which notification was sent was greater in the mSOS group than in the control group (27.3% vs 4.8%), but the difference was of borderline statistical significance (95% CI = -0.32 to 34.13). Similar differences were found when the analysis was restricted to health facilities that stocked paper-based tools (ie, control group, 1/18 (5.6%) vs mSOS group, 22/78 (22.6%); % difference 17.0, 95% CI = -2.93 to 35.30).	Low
Yugi (2016), Sudan, Low income [22]	Observational pre/post	The aim of the study was to evaluate a SMS based epidemiology data collection system for CHWs in Sudan. CHWs were trained in the use of a mobile platform-based epidemiology reporting system. Timeliness and completeness of the data report were evaluated before and after the implementation of the new system.	128 persons reported epidemiology data via SMS	Post intervention, all included facilities reported epidemiology data on time. The authors report 'increased on-time submissions upon introduction of the app.'	Very low
**Decision support:**					
Amoakoh (2019), Ghana, Lower-middle income [23]	Cluster RCT	A cluster RCT was performed in Eastern Ghana. Over 18 mo, 16 districts were randomized to receive mHealth support or not. Those that received support were evaluated for patterns of support received in the context of patient presentations and characteristics.	16 regions including hospitals were randomly divided into 8 experimental and 8 control clusters.	Maternal decision support was utilized more frequently than neonatal decision support (66.35% vs 33.65%; *P* < 0.001). There was a 20.02% (331/1653) and a 57.56% (761/1322) decline respectively, in the number of maternal requests made from the first to the second 6 months and from the second to the third 6 months of intervention implementation (*P* < 0.001).	Moderate
Bavdekar (2005), India, Lower-middle income [24]	Observational cohort	Using final clinical diagnosis as the gold standard in a tertiary pediatric center in India, the sensitivity of the ISABEL diagnostic tool, an Internet-delivered pediatric diagnosis support system, to make the final diagnosis was evaluated based on initial presentation.	Records of 200 subjects admitted to the Pediatric ICU (boys 111, girls 89, aged 28 days-12 years) were analyzed.	The diagnostic tool missed 27 diagnoses (for example: septicemia, tuberculosis and seizures) in 39 subjects providing a sensitivity of 80.5%.	Very low
Bilal (2018), Bangladesh, Lower-middle income [30]	Observational cohort	A prospective cohort of patients presenting with acute diarrhea to a tertiary care hospital were assessed by either using either the standard WHO algorithm printed on a laminated card or an mHealth-supported WHO algorithm downloaded onto a smartphone in order to assess the feasibility of an mHealth-supported WHO algorithm for the assessment of dehydration in patients with acute diarrhea in a rural, low-income country setting.	A total of 496 patients with acute diarrhea (<5 years, N = 349, >5 years, N = 147) were enrolled.	Of the 496 patients, 132 (27%) had some or severe dehydration, and 364 (73%) had no dehydration on arrival. Cohen’s K statistic demonstrated greater reliability for the mHealth-supported dehydration assessment (0.59) compared with the standard assessment (0.50) in the overall population (*P* < 0.0001), as well as in the pediatric (0.43 vs 0.37, *P* < 0.0001) and adult (0.79 vs 0.57, *P* < 0.0001) populations individually.	Low
Blom (2017), South Africa, Upper-middle income [25]	Observational cohort	In order to evaluate the accuracy of image-based remote burn diagnosis in emergency care in South Africa, a web-based questionnaire was created with 51 images of burns representing those cases. Participating burns specialists from two countries were asked to assess each burn’s total body surface area (TBSA) and depth using a smartphone or tablet as compared to the bedside assessment.	8 South African burn specialists, 7 Swedish burn specialists, and 11 South African Emergency medicine specialists were enrolled.	The assessments of TBSA are of high accuracy all specialists aggregated (ICC = 0.82 overall and 0.81 for both child and adult cases separately) and remain high for all three participant groups separately. The burn depth assessments have low accuracy all specialists aggregated, with ICCs of 0.53 overall, 0.61 for child and 0.46 for adult cases.	Low
Crehan (2019), Malawi, Low income [26]	Observational pre/post	An mHealth application (NeoTree) that provides evidence-based decision support for health workers in newborn care and resuscitation was deployed in a district hospital in Malawi. The authors conducted surveys focusing on usability before and after one month of clinical use.	46 health workers were enrolled.	Health workers report high usability scores before and after the study. They also reported high perceived improvements in quality of newborn care.	Very low
Duffy (2017), Uganda, Low income [27]	Observational cohort	To determine whether MedNav, an mHealth decision support and activity-prompting tool, improves neonatal resuscitation quality, it was employed in a Ugandan hospital, and using ideal resuscitation per the WHO as the gold standard, midwife resuscitation of neonates was evaluated with and without the aid of MedNav.	46 total resuscitations were included: 20 with MedNav and 26 without MedNav.	The adherence to good practice increased from a mean of 46% without MedNav to 94% with MedNav. The mean system usability score was self-assessed at 84.5%. Older staff less often approved of use of the MedNav app.	Very low
Finette (2019)	Observational cohort	This study describes the development and initial validation testing of a mobile health (mHealth) platform, MEDSINC, designed to provide frontline health workers (FLWs) with a validated clinical risk assessment tool for children aged 2-60 months. Across three different countries, clinical assessments by FLWs aided by MEDSINC were independently and blindly correlated with clinical assessments by 22 local health care professionals (LHPs).	861 children aged between 2 and 60 months were assessed via MEDSINC	Clinical assessments by FLWs using MEDSINC had a specificity correlation between 84% and 99% to LHPs. MEDSINC triage recommendation distributions were highly correlated with those of LHPs. Inter-rater reliability analysis revealed clinical assessments agreement between MEDSINC and LHP greater than would be expected because of chance. Usability and feasibility responses from LHP/FLW were collectively positive for ease of use, learning, and job performance.	Low
Burkina Faso, Low income					
Ecuador, Upper-middle income					
Bangladesh, Lower-middle income [14]					
Haque (2017), Bangladesh, Lower-middle income [28]	Observational pre/post	This is a pilot study which took place during the 2015 cholera season. WHO diarrheal guidelines were adopted to a smartphone platform and provided to clinicians. Clinical care was evaluated during a 6-week pre-intervention and 6-week intervention period with a 10-day post-discharge follow-up.	A total of 841 patients were enrolled (325 pre-intervention; 516 intervention).	During the intervention, the use of IV fluids decreased at the district and sub-district hospitals (both *P* < 0.001). When IV fluids were prescribed, the volume better adhered to recommendations. The proportion of prescriptions for the recommended antibiotic azithromycin increased (*P* < 0.001) while prescriptions for other antibiotics decreased. Zinc adherence increased.	Low
Savatmongkorngul (2017), Thailand, Upper-middle income [29]	Observational cohort	This study aims compared an mHealth emergency severity index (ESI) tool to the original, paper based ESI triage. Eligible patients presenting to an ED were evaluated using either the original or mobile ESI. The ESI results for each patient were compared with the standard ESI. Concordance and kappa statistics were calculated for pairs of the evaluators. An independent researcher applied the ESI to all cases as the gold standard.	486 patients enrolled in the study. 235 patients were assessed using the mobile ESI, and 251 patients were in the paper based ESI group.	The percentage of concordance and kappa statistics in the original ESI group were lower than those of the mobile group in all three comparisons (*P* < 0.01). Compared with the gold standard, the original ESI had higher rates of both under-triage and over-triage than the mobile ESI (p value <0.001 for medical students and 0.001 for emergency physicians).	Low
**Direct patient care:**					
Brangel (2018), Uganda, Low income [31]	Observational cohort	In order to evaluate a smartphone based, point-of-care test for Ebola IgG antibodies, an assay an app was produced and internally validated on previously obtained sera from Ebola survivors and subsequently underwent a pilot test on Ugandan Ebola survivors and matched controls.	Serum from 25 Ebola survivors and 5 controls were studied	The mHealth based assay demonstrated 100% sensitivity and 100% specificity when read with the smartphone application.	Low
Chauhan (2018), India, Lower-middle income [32]	Observational cohort	In order to evaluate the effectiveness of 24-h Tele-ECG support, eight rural community health centers were provided with 24-h Tele-ECG support. The times to diagnosis and treatment with anti-platelet agents were compared against health centers without Tele-ECG support	8 intervention and 6 control health centers were included.	The median hospital-to-aspirin time (h) in the intervention and the control groups was 0.7 ± 1.45 h and 3.5 ± 10 h, respectively (*P* < 0.0001). Aspirin was administered to 91% and 58% of patients with acute coronary syndrome in the intervention and the control groups, respectively (*P* < 0.0001). There was no difference in pain to aspirin time or thrombolysis rate.	Low
Chen (2016), China, Upper-middle income [33]	Observational pre/post	The before-and-after study studied whether implementation of a prehospital digital program for EKG transmission can reduce the door-to-balloon (D2B) time for percutaneous coronary intervention (PCI) in acute chest pain patients.	609 intervention vs 528 consecutive controls were included.	Patients achieved a D2B time <90 min using tele-EKG (82.5 vs 26.0%, *P* < 0.001). The intervention reduced D2B time and reduced hospitalization lengths and costs (all *P* < 0.001)	Low
Chinprasatsak (2017), Thailand, Upper-middle income [34]	RCT	Using real time video and audio data in for EMS providers, this RCT evaluated the impact on emergency physician support in prehospital diagnosis and interventions in advanced life support teams in urban Thailand.	100 random consecutive patients were enrolled and provided routine care or telegraphic prehospital assistance	Telegraphic medicine systems significantly increased the percentage of cases with primary diagnosis (*P* < 0.01). There were trends towards increasing the percentages of patients receiving appropriate airway, circulatory, and neurological management, but none were statistically significant.	Moderate
Dharmasaroja (2010), Thailand, Upper-middle income [35]	Observational pre/post	A pre/post style observational study was undertaken after the organization of the Thammasat Stroke Network (TSN). The main outcome measures included favorable outcome of the patients treated with intravenous tissue plasminogen activator (tPA) at 3 mo and symptomatic intracerebral hemorrhage by comparison between walk-in patients and the patients who were referred by the TSN.	170 acute stroke patients pre-TSN were included, and there were 406 in the post-TSN period.	14 patients (14 out of 170 acute ischemic stroke patients, 8%) and 110 patients (110 out of 406 patients, 27%) received tPA, before and after implementation of TSN, respectively. Walk-in patients (66 patients) had significant shorter onset-to-treatment duration as compared with referred patients (58 patients) (*P* < 0.0001). However, there was no significant difference in favorable outcome (48 vs 42%, *P* = 0.538). The rate of symptomatic intracerebral hemorrhage (3 vs 2%, *P* = 0.637).	Low
Filho (2018), Brazil, Upper-middle income [36]	Observational pre/post	This study described temporal trends in 30-d mortality and identified predictors of mortality among STEMI patients enrolled in a prospective registry in Brazil following implementation of a regional telemedicine-based STEMI network including 23 hospitals and their EMS providers. This network was initiated in January of 2011.	520 total subjects were enrolled and evaluated based on date of presentation	Overall mortality at 30 days was 15.0%. Use of dual antiplatelet therapy and statins increased significantly from baseline in January 2011 through June 2013: 61.8% to 93.6% (*P* < 0.001) and 60.4% to 79.7% (*P* < 0.001), respectively. Rates of primary reperfusion also increased (29.1%–53.8%; *P* < 0.001), and more patients were transferred to the referral center (44.7%-76.3%; *P* = 0.001). Thirty-day mortality rates decreased from 19.8% to 5.1% (*P* < 0.001).	Moderate
Garde (2016), Bangladesh, Lower-middle income [37]	Observational cohort	In order to develop a tool to identify children at high risk of hospital admission, a mobile app was utilized to collect 1-min segments of pulse oximetry, blood oxygen saturation (SpO2), heart rate, and respiratory rate in children under 5 years of age presenting to a tertiary care hospital. A univariate age-adjusted logistic regression was applied to evaluate differences between admitted and non-admitted children.	615 admitted children were compared to 1435 children not requiring admission.	Children admitted to hospital showed significantly (*P* < 0.01) decreased pule rate variability (PRV) and higher SpO2 variability compared to non-admitted children. The strongest predictors of hospitalization were reduced PRV-power in the low frequency band, greater time spent below an SpO2 of 98% and 94%, high respiratory rate, high HR, low SpO_2_, young age, and male sex.	Moderate
Ma (2019), Uganda, Low income [38]	Observational cohort	This is a prospective cohort study studying the predictive value of a handheld device point of care lactate in children <5 years admitted to two hospitals in Uganda with clinical signs of pneumonia. The cohort was followed through the course of their hospitalization with the primary outcome being lactate as a predictor of mortality.	Of the cohort of 150 patients, 22 subjects expired and 128 survived.	Median admission lactate level was 2.4 (1.8-3.6) mmol/L among children who survived vs 7.2 (2.6-9.7) mmol/L among those who died (*P* < 0.001). Lactate was a better prognostic marker of mortality (*P* < 0.001), than any single clinical sign or composite clinical risk score. Lactate level at admission of <2.0, 2.0-4.0, and >4.0 mmol/L accurately risk stratified children, with 5-d mortality of 2%, 11% and 26%, respectively (*P* < 0.001).	Moderate
Macedo (2016), Brazil, Upper-middle income [39]	Observational pre/post	This before-and-after study compared the use of a pharmacoinvasive strategy and mortality in patients with ST elevation myocardial infarction (STEMI) transferred pre– and post–implementation of a chest pain protocol with access to a 24 h telemedicine cardiologist support in a private hospital network in Brazil.	The authors enrolled 376 patients (113 pre-protocol and 263 post-protocol) with STEMI	The implementation of the STEMI protocol involving telemedicine cardiology support was associated with a greater use of pharmacoinvasive strategy in the care for STEMI (55.8% vs 38%; *P* = 0.002) and a non-significant trend toward lower in-hospital mortality (3% vs 8%; *P* = 0.06).	Low
Marcolino (2013), Brazil, Upper-middle income [40]	Observational pre/post	This before-and-after study assessed the 2010 initiation of an acute myocardial infarction (AMI) management protocol involving telehealth electrocardiogram support for hospital staff in a private Brazilian hospital network. The primary outcomes of this retrospective observational study were the number of admissions and in-hospital mortality due to AMI, from 2009 to 2011.	AMI patients by intervention phase: Before (2009): 1242; During (2010): 1113; Post (2011): 1358	A significant reduction was observed in the in-hospital mortality rate (12.3% in 2009 vs 7.1% in 2011, *P* < 0.001). The mean cost of admission increased (mean R$ 2480.00 vs R$ 3501.00; *P* < 0.001), the proportion of admissions including intensive care unit stay increased (32.4% in 2009 vs 66.1% in 2011; *P* < 0.001), and the number of patients admitted to tertiary hospitals increased (47.0% vs 69.6%; *P* < 0.001).	Low
Rahman (2017), Bangladesh, Lower-middle income [41]	Observational pre/post	This before-and-after study evaluated the effectiveness of an app-based Blood Information Management Application (BIMA) system for reducing lag time in the blood transfusion process in acutely ill patients. A median linear regression model was employed to assess the adjusted effect of BIMA on transfusion time.	The authors included 143 and 177 cases for the before and after phase of BIMA intervention, respectively.	After introducing BIMA and after adjusting for criteria such as maternal age, education, parity, duty roster of providers, and reasons for blood transfusion, a 24-min reduction in the time was observed between the identified need for blood and transfusion (*P* < 0.001).	Low
Saberian (2019), Iran, Upper-middle income [42]	Observational cohort	This study aimed to assess the role of pre-hospital triage via telecardiology on coronary reperfusion time of patients with ST segment elevation myocardial infarction (STEMI). Consecutively sampled patients were divided into two groups of percutaneous coronary intervention (PCI) following telecardiology or PCI following emergency department (ED) diagnosis of STEMI .	1205 total STEMI patients were included. 841 were transferred directly to the cardiac cath laboratory, and 364 were first admitted to the ED.	Symptom-to-device interval time in patients who underwent PCI following telecardiology was significantly lower (*P* < 0.001); however, the difference was not significant in the first medical contact (FMC)-to-device interval time (*P* = 0.268).	Very low
Steinhubl (2016), Sierra Leone, Low income [43]	Observational cohort	This was a three-week observational pilot study for the Modular Wireless Patient Monitoring System (MWPMS), designed for improved, automated patient oversight while limiting health care work risk in the setting of Ebola Virus Disease. The authors prospectively enrolled patients admitted to an Ebola Treatment Center (ETC) in order to compare a wireless, multiparametric ‘band-aid’ patch sensor for continuous vital sign monitoring and transmission.	26 subjects were enrolled. They received both the MWPMS and standard of care vital sign monitoring.	A total of 1838 h of continuous multiparametric waveform data were collected, with 91% of the data determined to be of good quality. The correlation between temperature measured by the infrared thermometer and the sensor patch was very strong (R = 0.99, *P* < 0.001), whereas the correlation for heart rate (R = 0.75, *P* < 0.001) and respiratory rate (R = 0.83, *P* < 0.001) were less strong but still robust.	Very low
Tirivayi (2016), Zimbabwe, Lower-middle income [44]	Observational cohort	The aim of the study was to evaluate an individual level mobile cash transfer program utilized by humanitarian aid agencies to offset food insecurity in drought circumstances in Zimbabwe in 2015. The project provided unconditional mobile cash transfers (MCT) to more than 65 000 vulnerable households in the drought-affected communities of the southern provinces of Zimbabwe. Simple random sampling was employed to obtain quantitative data via a structured household survey of beneficiary and non-beneficiary households.	A sample of 416 beneficiary households and 422 non-beneficiary households were randomly selected and interviewed in February 2016	Nearly 90% of the cash transfer was spent on food. The dietary diversity of beneficiaries increased by 0.3 units or 8% and self-reported hunger decreased by nearly 18%. Compared to non-beneficiaries, the probability of meeting the minimum acceptable diet was 15% higher among beneficiaries. There was a significant reduction in the proportion of households skipping eating for entire days. However, there was no significant reduction in other food rationing coping strategies. There were no significant impacts on the coping strategies index or on other extreme coping strategies such as school withdrawal or distress sale of assets.	Low
Zachariah (2012), Somalia, Low income [45]	Observational pre/post	The goal of this study was to assess the impact of introducing telemedicine on the quality of pediatric care in a health care facility within a war-torn region of Somalia. Medecins Sans Frontieres (MSF) implemented a telehealth program for acutely ill children requiring admission to the district hospital. A retrospective analysis of program data and a perception study among the local clinicians was undertaken.	2102 pediatric admissions in 2010 (prior to telemedicine) were included and 3873 admissions with access to telemedicine were included.	Adverse outcomes (deaths and lost to follow-up) decreased 30% from a total of 7.6% in 2010 to 5.4% in 2011 (OR = 0.70, *P* = 0.001). Formal referrals to a higher-level facility also significantly increased from 0.05 to 0.4% (OR = 7.6, *P* = 0.02). The number needed to be treated (NNT) through telemedicine to prevent one adverse outcome (death or loss to follow-up) was 45. All seven clinicians surveyed found telemedicine to be useful and frequently practice changing.	Low
**Health trainings:**					
Bolan (2018), Democratic Republic of the Congo, Low-income [46]	Cluster RCT	This trial randomized 8 health facilities in the DRC to receive app-based learning support for three months or not, using pre/post tests at all health facilities to measure its impact. Specifically, they deployed an evidence-based mLearning training tool, the Safe Delivery App (SDA), which focuses on maternal and newborn health.	4 of the 8 enrolled health facilities received the app. A total of 32 intervention health workers and 30 control health workers were enrolled.	The mean increase in knowledge scores from pre- to post-test was statistically greater in the intervention group (*P* < 0.001). Self-confidence scores significantly improved compared with those of controls after 3 months (*P* = 0.02).	Low
Carrillo-Larco (2015), Peru, Upper-middle income [47]	Observational cohort	To order develop and evaluate the impact of an educational intervention with the use of text messages (SMS) in a first aid course, text messages were utilized as supplementary educational materials in a two-month first aid course for second year medical students.	66 intervention and 66 control students were enrolled.	The intervention group obtained higher scores compared with the control group (PR = 4.82; 95% CI = 1.58 to 14.72).	Low
Castro (2014), Mexico, Upper-middle income [48]	Observational cohort	To evaluate the impact of the type of communication media (face-to-face, telephone, videoconference) and type of nursing protocol media (paper-based, electronic-based) on patient interaction, twelve nurses evaluated standardized patients in video recorded rooms using three different types of communication media and three different types of protocol media.	12 nurses evaluated standardized patients in 6 different combinations of communication and protocol media.	Consultation time and duration of eye contact was lower in electronic based protocol media than paper based (*P* = 0.007 and *P* = 0.049 respectively). There was no difference in ultimate patient navigation and recommendations based on communication or protocol media.	Very low
Jain (2010), India, Lower-middle income [49]	RCT	This is an RCT that compared gains in knowledge and skills in neonatal resuscitation between tele-education instruction and conventional classroom teaching. An identical, single day session was either presented in person or via a tele-education with an assistant. Pre- and post-tests were employed immediately before and after trainings.	48 nurses at a tertiary care facility: 26 tele-education & 22 classroom teaching	Training resulted in a significant and comparable gain in knowledge scores (*P* = 0.06) and skills scores (*P* = 0.62) in both the groups. The post-training knowledge scores were comparable in the two groups (*P* = 0.55). However, the post-training scores, adjusted for baseline knowledge scores, were statistically higher in the in-person group compared with the tele-education group (*P* = 0.00).	Low
Kovacevic (2019), Bosnia and Herzegovina, Upper-middle income [50]	Observational pre/post	This study evaluated the impact of a yearlong tele-education intervention on patient care in an ICU. Weekly, structured tele-education conferences were conducted between two US trained intensivists and local critical care physicians. ICU structure, processes, and outcomes were evaluated before and after the introduction of the tele-education intervention. Sixteen providers evaluated changes in the ICU structure and processes after the intervention.	667 ICU patients were included in the preceding year. 595 were included during the intervention, and 633 were enrolled in the year following the intervention.	The intervention was associated with statistically significant reduction in ICU (43% vs 27%) and hospital (51% vs 44%) mortality, length of stay (8.3 vs 3.6 d), and cost savings ($400 000 over 2 y). A high level of staff satisfaction was reported with the tele-education program.	Low
Lin (2013), Vietnam, Lower-middle income [51]	Observational pre/post	The is a multicenter, prospective, pre/post-test study that was conducted in 11 hospitals with the goal of determining if a brief training intervention and the use of a clinical decision support tool could improve clinician scores on 15 question, multiple choice exams covering common pediatric emergencies. The primary outcome measure was the mean percentage difference in physician scores between the pretest and posttest.	A convenience sample of 203 participants, each with a pre-test and a post-test.	The intervention was effective. The mean pretest, posttest, and improvement scores were 37% (95% CI = 35%-38%), 70% (95% CI = 68%-72%), and 33% (95% CI = 30%-36%), respectively, with *P* < 0.0001.	Low
Liu (2019), China, Upper-middle income [52]	Observational pre/post	This is a retrospective cohort study that utilizes pre/post-tests to evaluate the effectiveness of an app-based mobile training system vs the previous year of standard trainings for emergency nurses. The training completion rate and pass rate were compared with the control data.	A convenience sample of 124 nurses was enrolled.	The training completion rate increased from <60% to 100%. The passing rate was 100%. 92.5% considered that the mobile phone platform was more convenient than conventional training course; 89.7% believed it as an effective tool.	Very low
Lund (2016), Ethiopia, Low income [53]	Cluster RCT	This is a cluster-randomized clinical trial in 5 rural districts of Ethiopia that evaluated the effects of the Safe Delivery App (SDA) on perinatal survival and health care workers’ knowledge and skills in neonatal resuscitation. From 2013 to 2015, 3601 women in active labor were included at admission and followed up until 7 days after delivery to record perinatal mortality. Knowledge and skills in neonatal resuscitation were assessed at baseline and at 6 and 12 mo after the intervention Analyses were performed based on the intention-to-treat principle	73 health care facilities were randomized to the mobile phone intervention or to standard care (control), which included among 176 health care workers at the included facilities (87 intervention, 89 control).	Use of the SDA was associated with a nonsignificant lower perinatal mortality of 14 per 1000 births in intervention clusters compared with 23 per 1000 births in control clusters (odds ratio, 0.76; 95% CI = 0.32-1.81). The skill scores of intervention health care workers increased significantly compared with those of controls at 6 mo (mean difference, 6.04; 95% CI = 4.26-7.82) and 12 months (mean difference, 8.79; 95% CI = 7.14-10.45) from baseline. Knowledge scores also significantly improved in the intervention compared with the control group at 6 months (mean difference, 1.67; 95% CI = 1.02-2.32) and at 12 months (mean difference, 1.54; 95% CI = 0.98-2.09).	High
Mikrogianakis (2011), Botswana, Upper-middle income [54]	Observational pre/post	This study sought to determine if telesimulation via videoconferences could be used by pediatricians in Toronto, Ontario, Canada, to teach an intraosseous (IO) insertion technique to physicians in Botswana. Learner’s opinions and skills were evaluated. Before and after the curriculum, physicians completed knowledge and procedural assessments.	22 physicians with pre/post surveys and skill demonstrations were included.	The mean scores on pre- and post-multiple choice testing significantly increased for comfort and knowledge inserting IO needles (*P* < 0.01), familiarity with the EZ-IO infusion system (*P* < 0.01), and knowledge handling the IO equipment (*P* < 0.01). Postintervention, 95% of physicians felt more prepared to manage pediatric resuscitation.	Very low
Onan (2019), Turkey, Upper-middle income [55]	RCT	This study investigated the effectiveness of traditional Basic Life Support training vs technology based instructional methods to achieve learning objectives of Basic Life Support education in students. Eighty-three voluntary students were randomly allocated to theoretical (Group A), video-based (Group B), and PocketCPR app-assisted video-based instructions (Group C). Assessments were conducted in training and 1week later.	25 students were assigned to each of the three groups.	Based on observable BLS scores, Group C (app based) mean scores were higher than those of Group A (*P* = 0.000) and Group B (*P* = 0.000). In terms of student confidence gained, Group C mean scores were higher than those of Group A but not Group B.	Very low
Otu (2016), Nigeria, Lower-middle income [56]	Observational pre/post	The study consisted of quantitative cross-sectional surveys in selected health facilities, before and after using the Ebola awareness tutorial (EAT) software. This educational course comprised essential information on Ebola Virus Disease. Knowledge, attitude and practice (KAP) measurements of health workers were measured.	203 health workers enrolled in the study.	The study showed an 11% improvement in average knowledge levels between pre- and post-intervention scores with statistically significant differences (*P* < 0.05) recorded for questions concerning the transmission of the Ebola virus among humans, common symptoms of Ebola fever and whether Ebola fever was preventable. Non-significant trends included increases health worker willingness to use PPE and practice frequent surface disinfection.	Very low
Saberi (2017), Iran, Upper-middle income [57]	Observational pre/post	This is a before-and-after type study that studied the effects of public education through SMS on the time from symptom onset to hospital arrival in patients with AMI in Iran. The authors measured symptom onset to call for help times and symptom onset to hospital arrival times. In the pre-intervention phase, admitted patients with AMI were enrolled. Next, a SMS educational message was sent to all residents of Kashan, and patients presenting in three months following this intervention were enrolled.	106 patients prior to intervention and 25 patients post-intervention were enrolled	The onset-to-door time was significantly shorter in the intervention group than the control group (240.53 ± 156.60 min vs 291.70 ± 251.23 min, *P* = 0.003). Moreover, the onset-to-call time was significantly shorter in the intervention group than the control group (127.06 ± 202.62 min vs 44.32 ± 81.26 min, *P* = 0.002).	Very low
Terry (2019), Uganda, Low income [58]	Observational pre/post	The study assessed the effectiveness of remote feedback on the quality of emergency medicine point of care ultrasound (POCUS) examinations for emergency care providers (ECPs) in rural Uganda. ECPs received initial training and then subsequently worked independently with remote feedback. Quality was assessed on a previously published eight-point ordinal scale by a US-based expert sonographer.	There were 1153 ultrasound exams recorded and evaluated across the three arms: Initial training, independent study, and remote feedback (quality assessment)	An improvement in quality from 3.82 (95% CI = 3.32-4.32) to 4.68 (95% CI = 4.35-5.01) on an eight-point scale was noted over the course of the study. The sensitivity and specificity of FAST exam during the initial training period was 77.8 (95% CI = 59.2-83.0) and 98.5 (95% CI = 93.3-99.9). Sensitivity improved to 88% compared to independent, non-feedback months whereas specificity was unchanged.	Very low
Yigzaw (2019), Ethiopia, Low income [59]	Observational cohort	The aim of this quasi-experimental study was to evaluate whether a blended learning approach to emergency obstetric and newborn care services involving SMS and phone calls could be as effective as a conventional learning approach while reducing costs. Providers were assigned to blended learning (12 days of offsite training followed by daily SMS and weekly phone calls) or conventional learning (18 days of offsite training followed by a facility visit to mentor participants). Provider skills were assessed three months post-training with an Objective Structured Clinical Examination (OSCE).	75 participants were allocated to the conventional approach, while 78 were enrolled in the blended approach.	Knowledge scores were similar for the blended and conventional learning groups before training (58.5% vs 61.5%, *P* = 0.358) and three months post-training (74.7% vs 75.5% = 0.720), with no significant difference in gains made. Post-training skills scores were significantly higher for conventional than blended learning (85.8% vs 75.3%, *P* < 0.001). Training costs were lower for blended learning than conventional learning (US$1032 vs US$1648 per trainee).	Low

### Data collection

The 8 studies in this category include 4 that were undertaken during humanitarian crises: two during the 2010 Haiti earthquake and two during the 2014-2016 West African Ebola epidemic. The Haitian studies report on the effectiveness of mobile phone sim card data to both predict population movements and infectious outbreak pressure in humanitarian crisis settings [15,16]. Mobile-phone based models outperformed traditional large data sets, and the calculated infectious pressure at outbreak onset was significantly correlated with reported cholera cases during the first ten days of the epidemic. Population level epidemiological monitoring was improved in all four studies evaluating mHealth data collection tools in the setting of disease outbreaks, such as EVD or malaria. When compared to the standard paper method, there was improved timeliness of submission and data completeness [17,18,21,22]. Two observational studies found short messaging service (SMS) an effective means to obtain individual level symptom and health seeking data [19,20]. Specifically, individual-level SMS messaging was used to monitor EVD symptoms and found to have a strong positive linear correlation between cell phone syndromic surveillance and reported EVD suspect cases and deaths. Mobile health data collection interventions appear to be effective on population and individual levels as compared to traditional paper or door-to-door data collection methods.

### Decision support

These 10 studies include a single RCT and nine observational studies that evaluated smartphone-based triage tools, evidence-based smartphone algorithms, and provided diagnostic support from specialists. To assess the feasibility of decision support instruments, a mixed-methods study utilized self-assessments to evaluate a neonatal care decision support application. Healthcare workers (HCWs) reported improved clinical confidence and perceived improvements in the quality of newborn care [26]. Further, a before-and-after study with a similar neonatal resuscitation support application was employed in a Uganda labor and delivery unit. This study employed trained third party observers to evaluate neonatal resuscitation and found the mean adherence to the standards of neonatal resuscitation to increase from 46% pre-intervention to 94% post-intervention [27]. Mobile health applications for patient triage outperformed standard paper assessments in acutely ill children and adults. These studies found improved accuracy in the triage of general ED patients in Thailand, acutely dehydrated patients with diarrhea in Bangladesh, and in a multinational cohort of children <5 years of age as compared to the gold standard paper triage assessments [14,29,30]. Finally, a single study found somewhat limited utility of remote visual diagnostics in pediatric burn patients. Remote specialists had highly accurate total body surface area (TBSA) estimates but poor depth assessments [25]. All nine studies concluded that mobile health decision support interventions were an effective means to improve EC in LMICs.

### Direct patient care

The 15 studies in this category include a single RCT and 14 observational trials. Six observational studies evaluated novel technologies such as point-of-care (POC) testing [31,38], patient monitoring devices [37,43], or app-based health resource management [41,44]. A pilot study evaluated the ability of smartphone cameras coupled with an mHealth app to perform a POC assay for Ebola IgG antibodies. It found 100% sensitivity and 100% specificity on a cohort of 25 known positive subjects and 5 controls [31]. Utilizing a novel, stand-alone mobile instrument, a prospective cohort of 150 children <5 years of age requiring admission in Uganda found POC lactate testing to be a better prognostic marker of mortality, than any single clinical sign or composite clinical risk score [38]. A mHealth app studied in Bangladesh collected minute-long segments of pulse oximetry, blood oxygen saturation (SpO_2_), heart rate, and respiratory rate in children under 5 years of age presenting to a tertiary care hospital. This application found pulse rate variability and hypoxia to be the two strongest predictors of admission [37]. Another prospective cohort compared a novel patient monitor device in an Ebola Treatment Center to standard nurse taken vital signs and displayed strong correlations for temperature, heart rate, and respiratory rate [43]. Finally, an mHealth application produced a 24-minute reduction in the time was observed between the identified need for blood and transfusion, as compared to standard paper systems [41].

The remaining nine studies encompassed telehealth interventions that took place in all phases of care from patient homes to the prehospital arena to health facilities, including a single telehealth study in a CHE. Telecardiology support for HCWs, in particular, was a recurrent theme encountered in this sector of mHealth. Six observational studies evaluating tele-cardiology support in acute coronary syndrome for prehospital providers or other HCWs such as emergency or primary care providers demonstrated significantly improved rates of administration of aspirin (91% vs 58%), improved rates of primary reperfusion, faster door-to-balloon (D2B) time for STEMI (D2B time <90 minutes: 82.5 vs 26.0%), a reduction of in-hospital mortality rates (12.3% vs 7.1%), and a reduction in thirty-day mortality rates (19.8 vs 5.1%) [32,33,36,39,40,42]. The lone RCT in this subgroup randomized ambulances staffed with EMS providers to real-time telehealth support vs standard care for the rate of correct primary diagnosis in the prehospital arena. They demonstrated significant improvement in arriving at the correct prehospital diagnosis and demonstrated nonsignificant trends towards improved the percentage of patients receiving appropriate prehospital management [34].

### Health training

A total of 14 studies described the use mHealth technologies for an educational purpose. These include four RCTs and 10 observational studies. The most common primary outcome in this subgroup was HCW knowledge or skills improvement. This group of studies utilized pre- and post-tests in cohorts of HCWs to demonstrate that mHealth interventions are an effective means to improve HCW knowledge and skills in various emergency capacities such as neonatal resuscitation, point of care ultrasonography (POCUS), and EVD knowledge [46,47,49-56,58,59]. An RCT evaluated the impacts of an educational mHealth app on perinatal survival in addition to HCW knowledge and skills in 73 health facilities in Ethiopia. The authors found that use of the application was associated with a nonsignificant lower perinatal mortality of 14 per 1000 births in intervention clusters compared with 23 per 1000 births in control clusters but did find significant improvements in HCW skills and knowledge [53]. A similar mHealth intervention was applied in a public health context. This observational study on SMS-based education in lay persons in Iran found that educational messages on acute coronary syndrome symptoms decreases both the symptom onset to ED arrival and call for help times [54].

### Complex humanitarian emergency

Of all 46 included studies, 10 publications evaluated the role of mHealth in CHEs. Half of these were performed during the 2014 Ebola outbreak, two were performed during the 2010 Haitian earthquake, and three took place in the setting of civil unrest and profound food insecurity. Specific to the 2014 Ebola outbreak, an observational study demonstrated that an mHealth based educational program was an effective way to improve health worker knowledge on EVD [56]. Further observational publications demonstrated that SMS messaging was effective on the individual level for monitoring health care behaviors and symptom development, respectively [19,20,48]. Two publications demonstrated the utility of novel patient care devices, piloting the use of a wearable remote patient vital sign monitor and a point-of-care EVD IgG test respectively [31,43]. Post-Haitian earthquake, two large observational data sets demonstrated the utility of anonymous cell phone data in both tracking population movements and predicting regional infectious disease outbreaks based on these movements [15,16]. To evaluate the impact of mHealth on epidemic surveillance during a CHE, a before-and-after study demonstrated that mHealth tools result in significantly improved completeness and timeliness as compared to traditional paper-based methods [18]. A before-and-after study evaluated the impact of an Médecins Sans Frontiérs (MSF) telehealth intervention on patient care in a war-torn region of Somalia. Over two years, this study enrolled nearly 6000 acutely ill children requiring hospital admission and demonstrated that telehealth support from Kenyan pediatricians significantly decreased the risk of death or loss to follow up [45]. Finally, the impact of United Nations supported mobile cash transfer program on diet and hunger in drought-affected communities in Zimbabwe was evaluated prospectively via random household surveys. 90% of the cash transfers were spent on food, which improved dietary diversity, decreased self-reported, and significantly improved the probability of meeting the minimum acceptable dietary standards [44].

### Patient-centered outcomes

Twelve (26%) of the 46 total studies were patient-centered in nature. Six studies reported the impact of mHealth interventions on mortality. Four observational cohorts found their respective interventions to decrease mortality rates [36,40,45,50]. Two before-and-after observational trials evaluating regional telecardiology programs in Brazil found these system wide interventions significantly increased rates of primary reperfusion in STEMI (29.1 vs 53.8%) and ICU admission (32.4 vs 66.1%) while decreasing both thirty-day mortality rates (19.8 seconds, 5.1%) and in-hospital mortality rates (12.3 vs 7.1%) [36,40]. A yearlong ICU-based study evaluated weekly tele-education conferences focused on ICU structure, processes, and outcomes in Bosnia and Herzegovina. This before-and-after study enrolled approximately 600 patients yearly over 3 years and demonstrated statistically significant reductions in ICU mortality (43% vs 27%), in-hospital mortality (51% vs 44%), and length of stay (8.3 vs 3.6 days), over the study period [50]. The MSF supported before-and-after study described above demonstrated that telehealth support decreases mortality in pediatric patients requiring hospital admission in a CHE [45]. Two studies reported non-significant trends towards decreased mortality [39,53]. The lone RCT with patient centered outcomes evaluated the impacts of an educational application on perinatal mortality and did not demonstrate a statistically significant mortality benefit. Extrapolating that door-to-balloon (D2B) time and appropriate pharmacologic management of acute coronary syndrome (ACS) is associated with a reduced risk of death [60], three additional studies demonstrated favorable patient outcomes by proxy [32,33,57]. A Thailand based tele-stroke focused study demonstrated increased adherence to guideline-based use of tPA via a tele-stroke network but found no difference in favorable neurologic outcomes at 3 months [61].

## DISCUSSION

The studies included in this review demonstrated a positive impact of mHealth interventions on the quality of EC in LMICs. The measured outcomes are diverse and largely showed improvement of provider-focused and patient-centered outcomes regardless of setting or subset of mHealth intervention (data collection, decision support, direct patient care, or health training). These findings are in line with previous systematic reviews examining the impacts of mHealth in LMICs in disciplines outside of EC, such as chronic disease management [62-64].

The mHealth studies on data collection included in this systematic review demonstrated improved epidemic monitoring data quality and timeliness in both routine EC and complex humanitarian emergencies, such as the 2014-16 EVD outbreak, compared to traditional paper standard. On the individual level, mHealth applications may effectively monitor both symptomatology and health resource utilization. On the population level, mHealth interventions may effectively predict population movement and infectious disease outbreak risks, enabling public health officials to anticipate and intervene in potentially problematic regions.

Decision support studies report satisfactory clinical utility and reliability of a variety of decision support algorithms targeted for HCWs. These studies aimed at improving health care quality by supporting HCWs with evidence-based medicine algorithms; however, they focused primarily on usability or HCW satisfaction with their respective mHealth interventions. Two studies did report improved HCW adherence to established international treatment guidelines, which may improve clinical care by proxy [27,28].

Direct patient care was the most robust subset of mHealth studies included in this systematic review. Based on average GRADE scores, this subset has a higher percentage of quality publications. The majority were telehealth interventions, which demonstrated a high proportion of positive patient-centered outcomes. The tele-cardiology studies generally demonstrated improved patient outcomes and clinical care consistent with prior publications from HICs [65]. The lone tele-stroke study improved rates of tPA administration [66]. The remaining studies in this subset consistently demonstrated the utility of novel technologies in EC in resource limited settings. From novel patient monitors in EVD units [67] to advanced point-of-care diagnostics [37,38], these studies showcased mHealth as a means to bring a high-quality and equitable level of care to remote, resource-constrained environments.

Finally, each of the 14 included educational studies conclude that mHealth is an effective means of EC health training in LMICs. This cohort of studies demonstrated that educational content does not always require traditional face-to-face teaching to improve provider knowledge and quality of care. In fact, mHealth supplementation may be superior to traditional teaching [47] and improves course completion rates [52]. Further, it may even improve patient outcomes by helping to ensure the delivery of consistent and high-quality care while increasing population access to health services [50]. Importantly, the majority of these studies did include formal curricula. Taken as a whole, these studies suggest that mHealth is an effective means to support educational needs in resource limited settings.

Notably, patient centered outcomes were generally lacking. Only one quarter of the studies measured patient-centered outcomes, which highlights the need for an increased focus on the important downstream impacts of mHealth interventions in the future.

### Limitations

Publication bias is likely the largest limitation of the literature found in this systematic review. The lack of negative studies encountered, particularly when evaluating novel technologies in resource-limited settings, may imply that many negative studies simply are not being published. Additionally, several studies include mHealth interventions, such as telecardiology, as part of a larger systemic intervention, making it impossible to distinguish to isolate effect of the mHealth component on endpoints such as mortality. These studies potentially introduce unmeasured confounding covariates that may skew the actual relationship between mHealth and the primary outcomes.

Methodologically, this systematic review was challenging. For example, the concept of social media technically falls under the umbrella term mHealth; however, it is its own complex social phenomenon, was recently the subject of an exhaustive review [68], and was therefore considered beyond the scope of this study. Additionally, given the difficulty of achieving the highest quality research in unstable environments, the choice was made to include pre/post studies without formal external controls. As such, the conclusions of this systematic review will need to be validated with future, high quality investigations focusing on evaluation of scaled-up mHealth interventions and the increased use of randomized-controlled trial methodologies. Another limitation of this study is the heterogeneity of outcomes, which precluded a formal meta-analysis. One final weakness is the selection bias introduced by only including publications in English and Spanish.

## CONCLUSIONS

This systematic review found that mHealth is effective in improving provider-focused, process-driven, and patient-centered outcomes in both routine and complex EC settings in LMICs through ensuring the delivery of consistent and high-quality care while improving access to health services. It is an effective means to improve data collection, purvey decision support, provide direct patient care, and offer health trainings in EC in LMICs. Future research with an increased focus on patient-centered outcomes is needed to validate these findings.

## Additional material

Online Supplementary Document
