# Supplementary material for: The effects of mobile health on emergency care in low- and middle-income countries: A systematic review and narrative synthesis
Source: J Glob Health. 2021 Apr 3;11:04023. doi: 10.7189/jogh.11.04023 (PMC8021077; doi:10.7189/jogh.11.04023)
Supplement: Online Supplementary Document [file jogh-11-04023-s001.pdf]

Appendix S1: Protocol

2019-2020 Systematic Review Protocol

Title: The effects of mobile health on emergency care in low and middle income countries

Registration: PROSPERO (CRD42019151080)

Logistical Support: Global Emergency Medicine Literature Review (GEMLR) Group, Usher Institute of Population Health Sciences and Informatics, University of Edinburgh

Financial Support: None

Conflicts of Interest: None

INTRODUCTION:
Rationale:

In recent years, mobile phones have evolved from simple communication devices to smart phone platforms advanced capabilities that boast a diverse variety of health care applications such as: decision support, data collection, lab analysis, chronic disease management, patient monitoring, telehealth and even real-time surgical assistance. All of these applications fall under the inclusive term mobile health (m-Health), which has found broad applications and wide spread success in resource limited areas, particularly in low and middle income countries (LMICs)[2].

Mobile health is defined by the World Health Organization (WHO) as “medical and public health practice supported by mobile devices, such as mobile phones, patient monitoring devices, personal digital assistants, and other wireless devices.” [1] Mobile health is a subset of Electronic Health (e-Health), which encompasses all transference of health resources and health care by electronic means. M-Health focuses on decision support, data collection, direct patient care or telehealth, and health education. It permits professionals to bring the latest health care innovations to remote areas of the globe that have been limited by traditional civil infrastructure such as roads and power grids[69 70]. Smart phone technology has bypassed these infrastructure shortcomings, and now, for example, there are 38 million mobile phone subscriptions in Kenya, a country of 44 million people[71].

Critical Review of Relevant Literature:

Objectives

Worldwide, m-health has been demonstrated to have positive health impacts in the care for chronic diseases[63], as well as the care for acute diseases[72].

Specifically, our objective is to evaluate:

- The effects of mobile health decision support in emergency care in LMICs and in complex humanitarian emergencies

METHODS:
Eligibility Criteria:

Studies from all years in English, Spanish, or French evaluating acute care applications of mHealth will be included. See Table 1.

Criterion	Included	Excluded
**Population**	Acute care / Emergency care in LMICs<br><br>Emergency care provided in complex humanitarian emergencies<br>o Manmade disasters<br>o Natural disasters<br>o Terrorist attacks<br>o War or conflict settings<br>o Epidemics<br><br>Emergency obstetric care	Primary care<br>Chronic disease care<br>High income countries
**Intervention**	Mobile Health (mHealth)<br>• Data Collection (epidemiology)<br>• Decision Support<br>• Direct Patient Care (teleHealth)<br>• Health training	Electronic Health (E-health) interventions such as electronic medical records or automation / digitalization of health care infrastructure
**Control**	Required	No control
**Outcome**	Health outcomes<br>Educational outcomes<br>Other relevant emergency care outcomes	
**Study Type**	Randomized control trials, observational studies with a control group, observation studies with a pre/post design	Case reports, case series, opinion papers, descriptive papers
**Language**	English and Spanish	
**Year of Publication**	All	

Table 1. PICO style presentation of systematic review

World Bank country classification based on income is updated July 1 of every year. Since 2010, 15 countries have reclassified as 'High Income' countries, meaning that publications utilizing data obtained after country reclassification will be excluded. By year, the following countries have been reclassified as high income. 2018: Argentina, Croatia, and Panama. 2017: Palau. 2016: British Virgin Islands, Gibraltar, Nauru. 2013: Antigua and Barbuda, Chile, Latvia, Lithuania. 2012: St. Kitts and Nevis. 2011: Curacao and St. Martin.

Exclusion criteria:
1. Study not in English or Spanish
2. Study clearly irrelevant to our topic
3. Intervention is not in an emergent care environment

4. Intervention is not M-health: decision support, data collection, direct patient care, or health training
5. Outcomes are non-healthcare related outcomes such as economic/monetary outcomes, supply chain efficiency, management or process outcomes

Information Sources:

Literature sources will include PubMed, Embase, DisasterLit, OVID: Global Health (CABI), LILAC, SciELO, Web of Science, and the Global Index Medicus (WHO). Grey literature will be included as well via advanced google searches targeting organizations known to publish global emergency care literature including but not limited to the World Health Organization, Doctors Without Borders, International Committee of the Red Cross, and the International Rescue Committee.

Search Strategy:
Search terms were developed via literature review, use of current Cochrane/WHO guidelines, and expert opinion. Search terms are available by request and will be made available in a published appendix.

Study Records:
Data management:
Records will be managed via a common shared cloud space: Microsoft Excel via Dropbox.

Selection process:
Upon completion of the initial searches, two independent reviewers will screen titles and abstracts for inclusion into the full text screening phase. Two independent reviewers will subsequently perform in depth evaluation of each selected full text article for inclusion into the synthesis and review. A third reviewer will independently review all titles, abstracts, and full text articles and serve as a tie breaker in case of reviewer disagreement.

Data collection process:
Data will be extracted utilizing standardized forms with preset variables. This process will be done in duplicate with the lead author mitigating any discrepancies for quality assurance.

Data items:

Data items will include but not be limited to first author, year published, years conducted, country, world bank development index, study design, m-Health application, control group, number of participants, statistical tests, outcomes.

Outcomes and prioritization:

Risk of bias in individual studies:
The Grading of Recommendations Assessment, Development, and Evaluation (GRADE) approach will be utilized to assess strength of evidence and risk of bias.

Data Synthesis:
Based on the initial literature review and perceived quality of evidence, we anticipate that the data will be qualitatively synthesized. Should we find objective information to the contrary, quantitative synthesis will be considered and performed.

Roles of team members:
This systematic review is Tyler Winders' dissertation through the University of Edinburgh. As such, he will be fully responsible for performing every step of the systematic review independently. Other reviewers will serve as second reviewers for title and abstract screening and for full text screening, should the need arise. Several other team members will serve in advisory and mentor roles, as tradition of the GEMLR group. Additionally, Tyler Winders will create his Masters in Public Health dissertation piece entirely independently.

Assessment of potential ethical or other risks, limitations and/or difficulties

Study environments are challenging in LMICs, as well as in complex humanitarian emergencies. Thus, the choice was made to include pre/post studies without formal controls recognizing that this will potentially influence the overall quality of the entire systematic review, particularly the synthesis of included studies. This is a limitation of the systematic review that will permit the authors to highlight cutting edge innovations in challenging environments, and it may be ameliorated by considering these studies for analysis separately of those at lower risk for bias based on the GRADE criteria.

Appendix 2: Search Strategy

Search Strategy: The effects of mobile health on emergency care in low and middle income countries

PubMed:

- **Date of Download:**
- **2674 results as of 10.1.19**

Emergency Medicine + Disaster
"disasters"[MeSH] OR "disaster medicine"[MeSH] OR "volcanic eruptions"[MeSH] OR "disease outbreaks"[MeSH] OR "hemorrhagic fever, ebola"[MeSH] OR "cholera"[MeSH] OR "measles"[MeSH:noexp] OR "starvation"[MeSH] OR "radioactive hazard release"[MeSH] OR "warfare and armed conflicts"[MeSH] OR "terrorism"[MeSH] OR "refugees"[MeSH] OR "refugee camps"[MeSH] OR "emergency responders"[MeSH] OR "emergency medical services"[MeSH] OR "emergency treatment"[MeSH] OR "emergency medicine"[MeSH] OR "ambulances"[MeSH] OR "critical care"[MeSH] OR "shock"[MeSH] OR "sepsis"[MeSH] OR "wounds and injuries"[MeSH] OR "pregnancy complications"[MeSH] OR "disaster"[tw] OR "disasters"[tw] OR "avalanche"[tw] OR "avalanches"[tw] OR "blizzard"[tw] OR "blizzards"[tw] OR "cyclone"[tw] OR "cyclones"[tw] OR "cyclonic storm"[tw] OR "cyclonic storms"[tw] OR "drought"[tw] OR "droughts"[tw] OR "earthquake"[tw] OR "earthquakes"[tw] OR "famine"[tw] OR "famines"[tw] OR "flood"[tw] OR "floods"[tw] OR "flooding"[tw] OR "hurricane"[tw] OR "hurricanes"[tw] OR "landslide"[tw] OR "landslides"[tw] OR "land slide"[tw] OR "land slides"[tw] OR "mudslide"[tw] OR "mudslides"[tw] OR "mud slide"[tw] OR "mud slides"[tw] OR "rockslide"[tw] OR "rockslides"[tw] OR "rock slide"[tw] OR "rock slides"[tw] OR "tidalwave"[tw] OR "tidalwaves"[tw] OR "tidal wave"[tw] OR "tidal waves"[tw] OR "tsunami"[tw] OR "tsunamis"[tw] OR "tornado"[tw] OR "tornadoes"[tw] OR "tropical storm"[tw] OR "tropical storms"[tw] OR "typhoon"[tw] OR "typhoons"[tw] OR "wildfire"[tw] OR "wildfires"[tw] OR "wild fire"[tw] OR "wild fires"[tw] OR "forest fire"[tw] OR "forest fires"[tw] OR "volcanic eruption"[tw] OR "volcanic eruptions"[tw] OR "disease outbreak"[tw] OR "disease outbreaks"[tw] OR "epidemic"[tw] OR "epidemics"[tw] OR "pandemic"[tw] OR "pandemics"[tw] OR "hemorrhagic fever"[tw] OR "hemorrhagic fevers"[tw] OR "haemorrhagic fever"[tw] OR "haemorrhagic fevers"[tw] OR "ebola"[tw] OR "cholera"[tw] OR "choleras"[tw] OR "measles"[tw] OR "starvation"[tw] OR "nuclear accident"[tw] OR "nuclear accidents"[tw] OR "radiation accident"[tw] OR "radiation accidents"[tw] OR "war"[tw] OR "wars"[tw] OR "warfare"[tw] OR "conflict"[tw] OR "conflicts"[tw] OR "terrorist attack"[tw] OR "terrorist attacks"[tw] OR "terrorism"[tw] OR "refugee"[tw] OR "refugees"[tw] OR "asylum seeker"[tw] OR "asylum seekers"[tw] OR "displaced person"[tw] OR "displaced persons"[tw] OR "humanitarian"[tw] OR "humanitarians"[tw] OR "humanitarianism"[tw] OR "mass casualty"[tw] OR "mass casualties"[tw] OR "mass shooting"[tw] OR "mass shootings"[tw] OR "emergency responder"[tw] OR "emergency responders"[tw] OR "emergency doctor"[tw] OR "emergency doctors"[tw] OR "emergency clinician"[tw] OR "emergency clinicians"[tw] OR "emergency physician"[tw] OR "emergency physicians"[tw] OR "emergency personnel"[tw] OR "emergency medical personnel"[tw] OR "emergency service"[tw] OR "emergency services"[tw] OR "emergency medical service"[tw] OR "emergency medical services"[tw] OR "emergency medicine"[tw] OR "emergency health service"[tw] OR "emergency health services"[tw] OR "emergency care"[tw] OR "emergency healthcare"[tw] OR "emergency treatment"[tw] OR "emergency treatments"[tw] OR "emergency department"[tw] OR "emergency departments"[tw] OR "emergency room"[tw] OR "emergency rooms"[tw] OR "emergency ward"[tw] OR "emergency wards"[tw] OR "emergency unit"[tw] OR "emergency units"[tw] OR "emergency hospital"[tw] OR "emergency hospitals"[tw] OR "emergency clinic"[tw] OR "emergency clinics"[tw] OR "emergency setting"[tw] OR "emergency staff"[tw] OR "emergency response"[tw] OR "emergency medical technician"[tw] OR "emergency medical technicians"[tw] OR "paramedic"[tw] OR "paramedics"[tw] OR "ambulance"[tw] OR "ambulances"[tw] OR "ER"[tw] OR "first responder"[tw] OR "first responders"[tw] OR "rescue work"[tw] OR "rescue worker"[tw] OR "rescue workers"[tw] OR "relief work"[tw] OR "relief worker"[tw] OR "relief workers"[tw] OR "firefighter"[tw] OR "firefighters"[tw] OR "fire fighter"[tw] OR "fire fighters"[tw] OR "trauma center"[tw] OR "trauma centers"[tw] OR "trauma unit"[tw] OR "trauma units"[tw] OR "critical care"[tw] OR "critical illness"[tw] OR "critical illnesses"[tw] OR "resuscitation"[tw] OR "shock"[tw] OR "sepsis"[tw] OR "septicemia"[tw] OR "septicaemia"[tw] OR "acute care"[tw] OR "acute disease"[tw] OR "acute diseases"[tw] OR "prehospital"[tw] OR "pre hospital"[tw] OR "wound"[tw] OR "wounds"[tw] OR "triage"[tw] OR "pregnancy complication"[tw] OR "pregnancy complications"[tw] OR "obstetric complication"[tw] OR "obstetric complications"[tw] OR "obstetric

emergency"[tw] OR "obstetric emergencies"[tw]

Mobile Health

"cell phone"[MeSH] OR "cell phone use"[MeSH] OR "computers, handheld"[MeSH] OR "wireless technology"[MeSH] OR "mobile applications"[MeSH] OR "wearable electronic devices"[MeSH:noexp] OR "monitoring, physiologic"[MeSH:noexp] OR "telemedicine"[MeSH] OR "videoconferencing"[MeSH] OR "educational technology"[MeSH] OR "decision support systems, clinical"[MeSH] OR "mobile health"[tw] OR "mhealth"[tw] OR "m health"[tw] OR "cell phone"[tw] OR "cell phones"[tw] OR "cellular phone"[tw] OR "cellular phones"[tw] OR "cellular telephone"[tw] OR "cellular telephones"[tw] OR "wireless phone"[tw] OR "wireless phones"[tw] OR "wireless telephone"[tw] OR "wireless telephones"[tw] OR "mobile phone"[tw] OR "mobile phones"[tw] OR "mobile telephone"[tw] OR "mobile telephones"[tw] OR "smart phone"[tw] OR "smart phones"[tw] OR "smartphone"[tw] OR "smartphones"[tw] OR "smart device"[tw] OR "smart devices"[tw] OR "smart watch"[tw] OR "smart watches"[tw] OR "smartwatch"[tw] OR "smartwatches"[tw] OR "iPhone"[tw] OR "iPhones"[tw] OR "i Phone"[tw] OR "i Phones"[tw] OR "android"[tw] OR "blackberry"[tw] OR "mobile device"[tw] OR "mobile devices"[tw] OR "mobile communication"[tw] OR "mobile communications"[tw] OR "mobile technology"[tw] OR "mobile technologies"[tw] OR "mobile computing"[tw] OR "portable electronic device"[tw] OR "portable electronic devices"[tw] OR "mobile electronic device"[tw] OR "mobile electronic devices"[tw] OR "handheld device"[tw] OR "handheld devices"[tw] OR "hand held device"[tw] OR "hand held devices"[tw] OR "handheld computer"[tw] OR "handheld computers"[tw] OR "hand held computer"[tw] OR "hand held computers"[tw] OR "wireless device"[tw] OR "wireless devices"[tw] OR "wireless technology"[tw] OR "wireless technologies"[tw] OR "wireless monitor"[tw] OR "wireless monitors"[tw] OR "wireless monitoring"[tw] OR "wireless sensor"[tw] OR "wireless sensors"[tw] OR "personal digital assistant"[tw] OR "personal digital assistants"[tw] OR "tablet computer"[tw] OR "tablet computers"[tw] OR "computer tablet"[tw] OR "computer tablets"[tw] OR "iPad"[tw] OR "iPads"[tw] OR "i Pad"[tw] OR "i Pads"[tw] OR "iPod"[tw] OR "iPods"[tw] OR "i Pod"[tw] OR "i Pods"[tw] OR "app"[tw] OR "apps"[tw] OR "mobile application"[tw] OR "mobile applications"[tw] OR "portable software application"[tw] OR "portable software applications"[tw] OR "text message"[tw] OR "text messages"[tw] OR "text messaging"[tw] OR "texting"[tw] OR "short message service"[tw] OR "short message services"[tw] OR "short messaging service"[tw] OR "short messaging services"[tw] OR "SMS"[tw] OR "multimedia message"[tw] OR "multimedia messages"[tw] OR "multimedia messaging"[tw] OR "multi media messaging"[tw] OR "health message"[tw] OR "health messages"[tw] OR "health messaging"[tw] OR "wearable electronic device"[tw] OR "wearable electronic devices"[tw] OR "wearable computer"[tw] OR "wearable computers"[tw] OR "wearable device"[tw] OR "wearable devices"[tw] OR "wearable technology"[tw] OR "wearable technologies"[tw] OR "wearable sensor"[tw] OR "wearable sensors"[tw] OR "wearable monitor"[tw] OR "wearable monitors"[tw] OR "telemedicine"[tw] OR "tele medicine"[tw] OR "telehealth"[tw] OR "tele health"[tw] OR "telehealthcare"[tw] OR "tele healthcare"[tw] OR "telemonitoring"[tw] OR "tele monitoring"[tw] OR "teleeducation"[tw] OR "tele education"[tw] OR "teleconsult"[tw] OR "tele consult"[tw] OR "teleconsultation"[tw] OR "tele consultation"[tw] OR "telemanagement"[tw] OR "tele management"[tw] OR "telediagnosis"[tw] OR "tele diagnosis"[tw] OR "teleradiology"[tw] OR "tele radiology"[tw] OR "remote monitor"[tw] OR "remote monitoring"[tw] OR "remote consulting"[tw] OR "remote consultation"[tw] OR "remote consultations"[tw] OR "remote diagnosis"[tw] OR "remote education"[tw] OR "videoconference"[tw] OR "video conference"[tw] OR "videoconferencing"[tw] OR "video conferencing"[tw] OR "webcast"[tw] OR "webcasts"[tw] OR "webcasting"[tw] OR "clinical decision support"[tw]

LMIC: based on **Cochrane Foundation PubMed Filter**

Developing Countries[MeSH] OR Africa[MeSH] OR Asia[MeSH:noexp] OR Asia, Central[MeSH] OR Asia, Southeastern[MeSH] OR Asia, Western[MeSH] OR Caribbean Region[MeSH] OR South America[MeSH] OR Latin America[MeSH] OR Central America[MeSH] OR Afghanistan[MeSH] OR Albania[MeSH] OR Algeria[MeSH] OR American Samoa[MeSH] OR Angola[MeSH] OR "Antigua and Barbuda"[MeSH] OR Argentina[MeSH] OR Armenia[MeSH] OR Azerbaijan[MeSH] OR Bahrain[MeSH] OR Bangladesh[MeSH] OR Barbados[MeSH] OR Benin[MeSH] OR Byelarus[MeSH] OR Belize[MeSH] OR Bhutan[MeSH] OR

Bolivia[MeSH] OR Bosnia-Herzegovina[MeSH] OR Botswana[MeSH] OR Brazil[MeSH] OR Bulgaria[MeSH] OR Burkina Faso[MeSH] OR Burundi[MeSH] OR Cambodia[MeSH] OR Cameroon[MeSH] OR Cape Verde[MeSH] OR Central African Republic[MeSH] OR Chad[MeSH] OR Chile[MeSH] OR China[MeSH] OR Colombia[MeSH] OR Comoros[MeSH] OR Congo[MeSH] OR Costa Rica[MeSH] OR Cote d'Ivoire[MeSH] OR Croatia[MeSH] OR Cuba[MeSH] OR Cyprus[MeSH] OR Czechoslovakia[MeSH] OR Czech Republic[MeSH] OR Slovakia[MeSH] OR Djibouti[MeSH] OR "Democratic Republic of the Congo"[MeSH] OR Dominica[MeSH] OR Dominican Republic[MeSH] OR East Timor[MeSH] OR Ecuador[MeSH] OR Egypt[MeSH] OR El Salvador[MeSH] OR Eritrea[MeSH] OR Estonia[MeSH] OR Ethiopia[MeSH] OR Fiji[MeSH] OR Gabon[MeSH] OR Gambia[MeSH] OR "Georgia (Republic)"[MeSH] OR Ghana[MeSH] OR Greece[MeSH] OR Grenada[MeSH] OR Guatemala[MeSH] OR Guinea[MeSH] OR Guinea-Bissau[MeSH] OR Guam[MeSH] OR Guyana[MeSH] OR Haiti[MeSH] OR Honduras[MeSH] OR Hungary[MeSH] OR India[MeSH] OR Indonesia[MeSH] OR Iran[MeSH] OR Iraq[MeSH] OR Jamaica[MeSH] OR Jordan[MeSH] OR Kazakhstan[MeSH] OR Kenya[MeSH] OR Korea[MeSH] OR Kosovo[MeSH] OR Kyrgyzstan[MeSH] OR Laos[MeSH] OR Latvia[MeSH] OR Lebanon[MeSH] OR Lesotho[MeSH] OR Liberia[MeSH] OR Libya[MeSH] OR Lithuania[MeSH] OR Macedonia[MeSH] OR Madagascar[MeSH] OR Malaysia[MeSH] OR Malawi[MeSH] OR Mali[MeSH] OR Malta[MeSH] OR Mauritania[MeSH] OR Mauritius[MeSH] OR Mexico[MeSH] OR Micronesia[MeSH] OR Middle East[MeSH] OR Moldova[MeSH] OR Mongolia[MeSH] OR Montenegro[MeSH] OR Morocco[MeSH] OR Mozambique[MeSH] OR Myanmar[MeSH] OR Namibia[MeSH] OR Nepal[MeSH] OR Netherlands Antilles[MeSH] OR New Caledonia[MeSH] OR Nicaragua[MeSH] OR Niger[MeSH] OR Nigeria[MeSH] OR Oman[MeSH] OR Pakistan[MeSH] OR Palau[MeSH] OR Panama[MeSH] OR Papua New Guinea[MeSH] OR Paraguay[MeSH] OR Peru[MeSH] OR Philippines[MeSH] OR Poland[MeSH] OR Portugal[MeSH] OR Puerto Rico[MeSH] OR Romania[MeSH] OR Russia[MeSH] OR "Russia (Pre-1917)"[MeSH] OR Rwanda[MeSH] OR "Saint Kitts and Nevis"[MeSH] OR Saint Lucia[MeSH] OR "Saint Vincent and the Grenadines"[MeSH] OR Samoa[MeSH] OR Saudi Arabia[MeSH] OR Senegal[MeSH] OR Serbia[MeSH] OR Montenegro[MeSH] OR Seychelles[MeSH] OR Sierra Leone[MeSH] OR Slovenia[MeSH] OR Sri Lanka[MeSH] OR Somalia[MeSH] OR South Africa[MeSH] OR Sudan[MeSH] OR Suriname[MeSH] OR Swaziland[MeSH] OR Syria[MeSH] OR Tajikistan[MeSH] OR Tanzania[MeSH] OR Thailand[MeSH] OR Togo[MeSH] OR Tonga[MeSH] OR "Trinidad and Tobago"[MeSH] OR Tunisia[MeSH] OR Turkey[MeSH] OR Turkmenistan[MeSH] OR Uganda[MeSH] OR Ukraine[MeSH] OR Uruguay[MeSH] OR USSR[MeSH] OR Uzbekistan[MeSH] OR Vanuatu[MeSH] OR Venezuela[MeSH] OR Vietnam[MeSH] OR Yemen[MeSH] OR Yugoslavia[MeSH] OR Zambia[MeSH] OR Zimbabwe[MeSH] OR "developing country"[tw] OR "developing countries"[tw] OR "developing nation"[tw] OR "developing nations"[tw] OR "developing population"[tw] OR "developing populations"[tw] OR "developing world"[tw] OR "less developed country"[tw] OR "less developed countries"[tw] OR "less developed nation"[tw] OR "less developed nations"[tw] OR "less developed population"[tw] OR "less developed populations"[tw] OR "less developed world"[tw] OR "lesser developed country"[tw] OR "lesser developed countries"[tw] OR "lesser developed nation"[tw] OR "lesser developed nations"[tw] OR "lesser developed population"[tw] OR "lesser developed populations"[tw] OR "lesser developed world"[tw] OR “least developed country”[tw] OR “least

developed countries”[tw] OR “least developed nation”[tw] OR “least developed nations”[tw] OR “least developed population”[tw] OR “least developed populations”[tw] OR ”least developed world”[tw] OR "under developed country"[tw] OR "under developed countries"[tw] OR "under developed nation"[tw] OR "under developed nations"[tw] OR "under developed population"[tw] OR "under developed populations"[tw] OR "under developed world"[tw] OR "underdeveloped country"[tw] OR "underdeveloped countries"[tw] OR "underdeveloped nation"[tw] OR "underdeveloped nations"[tw] OR "underdeveloped population"[tw] OR "underdeveloped populations"[tw] OR "underdeveloped world"[tw] OR "middle income country"[tw] OR "middle income countries"[tw] OR "middle income nation"[tw] OR "middle income nations"[tw] OR "middle income population"[tw] OR "middle income populations"[tw] OR "low income country"[tw] OR "low income countries"[tw] OR "low income nation"[tw] OR "low income nations"[tw] OR "low income population"[tw] OR "low income populations"[tw] OR "lower income country"[tw] OR "lower income countries"[tw] OR "lower income nation"[tw] OR "lower income nations"[tw] OR "lower income population"[tw] OR "lower income populations"[tw] OR "underserved country"[tw] OR "underserved countries"[tw] OR "underserved nation"[tw] OR "underserved nations"[tw] OR "underserved population"[tw] OR "underserved populations"[tw] OR "underserved world"[tw] OR "under served country"[tw] OR "under served countries"[tw] OR "under served nation"[tw] OR "under served nations"[tw] OR "under served population"[tw] OR "under served populations"[tw] OR "under served world"[tw] OR "deprived country"[tw] OR "deprived countries"[tw] OR "deprived nation"[tw] OR "deprived nations"[tw] OR "deprived population"[tw] OR "deprived populations"[tw] OR "deprived world"[tw] OR "poor country"[tw] OR "poor countries"[tw] OR "poor nation"[tw] OR "poor nations"[tw] OR "poor population"[tw] OR "poor populations"[tw] OR "poor world"[tw] OR "poorer country"[tw] OR "poorer countries"[tw] OR "poorer nation"[tw] OR "poorer nations"[tw] OR "poorer population"[tw] OR "poorer populations"[tw] OR "poorer world"[tw] OR "developing economy"[tw] OR "developing economies"[tw] OR "less developed economy"[tw] OR "less developed economies"[tw] OR "lesser developed economy"[tw] OR "lesser developed economies"[tw] OR "under developed economy"[tw] OR "under developed economies"[tw] OR "underdeveloped economy"[tw] OR "underdeveloped economies"[tw] OR "middle income economy"[tw] OR "middle income economies"[tw] OR "low income economy"[tw] OR "low income economies"[tw] OR "lower income economy"[tw] OR "lower income economies"[tw] OR "low gdp"[tw] OR "low gnp"[tw] OR "low gross domestic"[tw] OR "low gross national"[tw] OR "lower gdp"[tw] OR "lower gnp"[tw] OR "lower gross domestic"[tw] OR "lower gross national"[tw] OR lmic[tw] OR lmics[tw] OR "third world"[tw] OR "lami country"[tw] OR "lami countries"[tw] OR "transitional country"[tw] OR "transitional countries"[tw] OR Africa[tw] OR Asia[tw] OR Caribbean[tw] OR West Indies[tw] OR South America[tw] OR Latin America[tw] OR Central America[tw] OR Afghanistan[tw] OR Albania[tw] OR Algeria[tw] OR Angola[tw] OR Antigua[tw] OR Barbuda[tw] OR Argentina[tw] OR Armenia[tw] OR Armenian[tw] OR Aruba[tw] OR Azerbaijan[tw] OR Bahrain[tw] OR Bangladesh[tw] OR Barbados[tw] OR Benin[tw] OR Byelarus[tw] OR Byelorussian[tw] OR Belarus[tw] OR Belorussian[tw] OR Belorussia[tw] OR Belize[tw] OR Bhutan[tw] OR Bolivia[tw] OR Bosnia[tw] OR Herzegovina[tw] OR Hercegovina[tw] OR Botswana[tw] OR Brasil[tw] OR Brazil[tw] OR Bulgaria[tw] OR Burkina Faso[tw] OR Burkina Fasso[tw] OR Upper Volta[tw] OR Burundi[tw] OR Urundi[tw] OR Cambodia[tw] OR Khmer Republic[tw] OR Kampuchea[tw] OR Cameroon[tw] OR Cameroons[tw] OR Cameron[tw] OR

Camerons[tw] OR Cape Verde[tw] OR Central African Republic[tw] OR Chad[tw] OR Chile[tw] OR China[tw] OR Colombia[tw] OR Comoros[tw] OR Comoro Islands[tw] OR Comores[tw] OR Mayotte[tw] OR Congo[tw] OR Zaire[tw] OR Costa Rica[tw] OR Cote d'Ivoire[tw] OR Ivory Coast[tw] OR Croatia[tw] OR Cuba[tw] OR Cyprus[tw] OR Czechoslovakia[tw] OR Czech Republic[tw] OR Slovakia[tw] OR Slovak Republic[tw] OR Djibouti[tw] OR French Somaliland[tw] OR Dominica[tw] OR Dominican Republic[tw] OR East Timor[tw] OR East Timur[tw] OR Timor Leste[tw] OR Ecuador[tw] OR Egypt[tw] OR United Arab Republic[tw] OR El Salvador[tw] OR Eritrea[tw] OR Estonia[tw] OR Ethiopia[tw] OR Fiji[tw] OR Gabon[tw] OR Gabonese Republic[tw] OR Gambia[tw] OR Gaza[tw] OR Georgia Republic[tw] OR Georgian Republic[tw] OR Ghana[tw] OR Gold Coast[tw] OR Greece[tw] OR Grenada[tw] OR Guatemala[tw] OR Guinea[tw] OR Guam[tw] OR Guiana[tw] OR Guyana[tw] OR Haiti[tw] OR Honduras[tw] OR Hungary[tw] OR India[tw] OR Maldives[tw] OR Indonesia[tw] OR Iran[tw] OR Iraq[tw] OR Isle of Man[tw] OR Jamaica[tw] OR Jordan[tw] OR Kazakhstan[tw] OR Kazakh[tw] OR Kenya[tw] OR Kiribati[tw] OR Korea[tw] OR Kosovo[tw] OR Kyrgyzstan[tw] OR Kirghizia[tw] OR Kyrgyz Republic[tw] OR Kirghiz[tw] OR Kirgizstan[tw] OR "Lao PDR"[tw] OR Laos[tw] OR Latvia[tw] OR Lebanon[tw] OR Lesotho[tw] OR Basutoland[tw] OR Liberia[tw] OR Libya[tw] OR Lithuania[tw] OR Macedonia[tw] OR Madagascar[tw] OR Malagasy Republic[tw] OR Malaysia[tw] OR Malaya[tw] OR Malay[tw] OR Sabah[tw] OR Sarawak[tw] OR Malawi[tw] OR Nyasaland[tw] OR Mali[tw] OR Malta[tw] OR Marshall Islands[tw] OR Mauritania[tw] OR Mauritius[tw] OR Agalega Islands[tw] OR Mexico[tw] OR Micronesia[tw] OR Middle East[tw] OR Moldova[tw] OR Moldovia[tw] OR Moldovian[tw] OR Mongolia[tw] OR Montenegro[tw] OR Morocco[tw] OR Ifni[tw] OR Mozambique[tw] OR Myanmar[tw] OR Myanma[tw] OR Burma[tw] OR Namibia[tw] OR Nepal[tw] OR Netherlands Antilles[tw] OR New Caledonia[tw] OR Nicaragua[tw] OR Niger[tw] OR Nigeria[tw] OR Northern Mariana Islands[tw] OR Oman[tw] OR Muscat[tw] OR Pakistan[tw] OR Palau[tw] OR Palestine[tw] OR Panama[tw] OR Paraguay[tw] OR Peru[tw] OR Philippines[tw] OR Philipines[tw] OR Phillipines[tw] OR Phillippines[tw] OR Poland[tw] OR Portugal[tw] OR Puerto Rico[tw] OR Rhodesia[tw] OR Romania[tw] OR Rumania[tw] OR Roumania[tw] OR Russia[tw] OR Russian[tw] OR Rwanda[tw] OR Ruanda[tw] OR Saint Kitts[tw] OR St Kitts[tw] OR Nevis[tw] OR Saint Lucia[tw] OR St Lucia[tw] OR Saint Vincent[tw] OR St Vincent[tw] OR Grenadines[tw] OR Samoa[tw] OR Samoan Islands[tw] OR Navigator Island[tw] OR Navigator Islands[tw] OR Sao Tome[tw] OR Saudi Arabia[tw] OR Senegal[tw] OR Serbia[tw] OR Montenegro[tw] OR Seychelles[tw] OR Sierra Leone[tw] OR Slovenia[tw] OR Sri Lanka[tw] OR Ceylon[tw] OR Solomon Islands[tw] OR Somalia[tw] OR Sudan[tw] OR Suriname[tw] OR Surinam[tw] OR Swaziland[tw] OR Syria[tw] OR Tajikistan[tw] OR Tadzhikistan[tw] OR Tadjikistan[tw] OR Tadzhik[tw] OR Tanzania[tw] OR Thailand[tw] OR Togo[tw] OR Togolese Republic[tw] OR Tonga[tw] OR Trinidad[tw] OR Tobago[tw] OR Tunisia[tw] OR Turkey[tw] OR Turkmenistan[tw] OR Turkmen[tw] OR Uganda[tw] OR Ukraine[tw] OR Uruguay[tw] OR USSR[tw] OR Soviet Union[tw] OR Union of Soviet Socialist Republics[tw] OR Uzbekistan[tw] OR Uzbek OR Vanuatu[tw] OR New Hebrides[tw] OR Venezuela[tw] OR Vietnam[tw] OR Viet Nam[tw] OR West Bank[tw] OR Yemen[tw] OR Yugoslavia[tw] OR Zambia[tw] OR Zimbabwe[tw]

OVID, Global Health (CABI):

- **Date of Download: 10.1.19**
- **N= 639**

Given same search engine utilized, Identical terms as Embase were employed.

Embase:

- **Date of Download: 10.1.19**
- **N= 2131**

Disaster/ OR Disaster medicine/ OR biological accident/ OR chemical accident/ OR nuclear accident/ OR avalanche/ OR drough/ OR earthquake/ OR flooding/ OR hurricane/ OR landslide/ OR tsunami/ OR volcano/ OR wildfire/ OR epidemic/ OR pandemic/ OR ebola hemorrhagic fever/ OR cholera/ OR measles/ OR food deprivation/ OR terrorism/ OR war/ OR warfare/ OR refugee/ OR refugee campe/ OR rescue personnel/ OR fire fighter/ OR ambulance/ OR emergency health service/ OR emergency treatment/ OR emergency care/ OR emergency medicine/ OR emergency ward/ OR resuscitation/ OR shock/ OR sepsis/ OR obstetric emergency/ OR labor complications/ OR disaster*.tw OR avalanche*.tw OR blizzard*.tw OR cyclone*.tw OR cyclonic storm*.tw OR drought*.tw OR earthquake*.tw OR famine.tw OR flood*.tw OR hurricane*.tw OR landslide*.tw OR mudslide*.tw OR tidal wave*.tw OR tsunami*.tw OR tornado*.tw OR typhoon*.tw OR tropical storm*.tw OR wild fire*.tw OR forest fire*.tw OR volcanic eruption*.tw OR disease outbreak*.tw OR epidemic*.tw OR pandemic*.tw OR hemorrhagic fever*.tw OR ebola.tw OR cholera.tw OR measles.tw OR starvation.tw OR nuclear accident.tw OR war*.tw OR conflict*.tw OR terrorist attack.tw OR refugee*.tw OR refugee camp*.tw OR displaced person.tw OR paramedic*.tw OR ambulance*.tw OR emergency medicine.tw OR ER.tw OR emergency department.tw OR first responder*.tw OR firefighter*.tw OR fire fighter*.tw OR critical care.tw OR shock.tw OR sepsis.tw OR acute care.tw OR prehospital care.tw OR obstetric complication.tw OR obstetric emergenc*.tw
telemedicine/ OR mobile phone/ OR cell phone/ OR personal digital assistant/ OR wireless communication/ OR wearable computer/ OR videoconferencing/ OR text messaging/ OR physiologic monitoring/ OR educational technology/ OR clinical decision support system/ OR mobile health.tw OR mhealth.tw OR m-health.tw OR text messag*.tw OR short message service.tw OR sms.tw OR mobile technology.tw OR mobile communication.tw OR mobile phone.tw OR smart phone.tw OR mobile device.tw OR cell phone.tw OR cellular telephone.tw OR portable electronic device.tw OR phone.tw OR tablet.tw OR PDA.tw OR ipod.tw OR android.tw OR blackberry.tw OR handheld device.tw OR handheld computer.tw OR hand held device.tw OR hand held computer.tw OR ipad.tw OR iphone.tw OR smartphone.tw OR mobile application.tw OR telemedicine.tw OR remote consult*.tw OR telemonitoring.tw OR remote monitor*.tw OR tele-education.tw OR educaitonal technology.tw OR decision support.tw OR remote diagnosis.tw OR telediagnosis.tw OR videoconferenc*.tw OR teleconsult.tw OR telehealth.tw OR teleradiology.tw OR clinical decision support.tw OR videoconferenc*.tw
developing country/ or low income country/ or middle income country/ or developing countr*.tw OR developing nation.tw OR middle income countr*.tw OR low income country*.tw OR global medicine.tw OR third world.tw OR underserved countr*.tw OR poor countr*.tw OR resource limited country.tw OR lmic*.tw OR low income economy.tw OR middle income economy.tw OR underdeveloped countr*.tw OR underdeveloped economy.tw OR poor countr*.tw OR poor nation.tw OR world health.tw OR low-income countr*.tw OR middle-income countr*.tw OR transitional countr*.tw OR lower middle income countr*.tw OR upper middle income.tw OR less developed countr*.tw OR lesser developed countr*.tw OR developing countr*.tw OR developing nation.tw OR lower-middle income countr*.tw OR upper-middle income countr*.tw OR low-income countr*.tw OR deprived countr*.tw OR low gdp.tw OR lami countr*.tw OR poorer nation.tw OR under served countr*.tw OR under served nation.tw OR lower income population.tw OR low income population.tw OR developing world .tw OR Africa.tw OR Asia.tw OR Caribbean.tw OR West Indies.tw OR South America.tw OR Latin America.tw OR Central America.tw OR Afghanistan.tw OR Albania.tw OR Algeria.tw OR Angola.tw OR Antigua.tw OR Barbuda.tw OR Argentina.tw OR Armenia.tw OR Armenian.tw OR Aruba.tw OR Azerbaijan.tw OR Bahrain.tw OR Bangladesh.tw OR Barbados.tw OR Benin.tw OR Byelarus.tw OR Byelorussian.tw OR Belarus.tw OR Belorussian.tw OR Belorussia.tw OR Belize.tw OR Bhutan.tw OR Bolivia.tw OR Bosnia.tw OR Herzegovina.tw OR Hercegovina.tw OR Botswana.tw OR Brasil.tw OR Brazil.tw OR Bulgaria.tw OR Chad.tw OR Chile.tw OR China.tw OR Colombia.tw OR Comoros.tw OR Comoro Islands.tw OR Comores.tw OR Mayotte.tw OR Congo.tw OR Zaire.tw OR Costa Rica.tw OR Cote d'Ivoire.tw OR Ivory Coast.tw OR Croatia.tw OR Cuba.tw OR Cyprus.tw OR Czechoslovakia.tw OR Czech Republic.tw OR Slovakia.tw OR Slovak Republic.tw OR Djibouti.tw OR French Somaliland.tw OR Dominica.tw OR Dominican Republic.tw OR East Timor.tw OR East Timur.tw OR Timor Leste.tw OR Ecuador.tw OR Egypt.tw OR United Arab Republic.tw OR El Salvador.tw OR Eritrea.tw OR Estonia.tw OR Ethiopia.tw OR Fiji.tw OR Gabon.tw OR Gabonese Republic.tw OR Gambia.tw OR Gaza.tw OR Georgia Republic.tw OR Georgian Republic.tw OR Ghana.tw OR Gold Coast.tw OR Greece.tw OR Grenada.tw OR Guatemala.tw OR Guinea.tw OR Guam.tw OR Guiana.tw OR Guyana.tw OR Haiti.tw OR Honduras.tw OR Hungary.tw OR India.tw OR Maldives.tw OR Indonesia.tw OR Iran.tw OR Iraq.tw OR Isle of Man.tw OR Jamaica.tw OR Jordan.tw OR

Kazakhstan.tw OR Kazakh.tw OR Kenya.tw OR Kiribati.tw OR Korea.tw OR Kosovo.tw OR Kyrgyzstan.tw OR Kirghizia.tw OR Kyrgyz Republic.tw OR Kirghiz.tw OR Kirgizstan.tw OR Laos.tw OR Latvia.tw OR Lebanon.tw OR Lesotho.tw OR Basutoland.tw OR Liberia.tw OR Libya.tw OR Lithuania.tw OR Macedonia.tw OR Madagascar.tw OR Malagasy Republic.tw OR Malaysia.tw OR Malaya.tw OR Malay.tw OR Sabah.tw OR Sarawak.tw OR Malawi.tw OR Nyasaland.tw OR Mali.tw OR Malta.tw OR Marshall Islands.tw OR Mauritania.tw OR Mauritius.tw OR Agalega Islands.tw OR Mexico.tw OR Micronesia.tw OR Middle East.tw OR Moldova.tw OR Moldovia.tw OR Moldovian.tw OR Mongolia.tw OR Montenegro.tw OR Morocco.tw OR Ifni.tw OR Mozambique.tw OR Myanmar.tw OR Myanma.tw OR Burma.tw OR Namibia.tw OR Nepal.tw OR Netherlands Antilles.tw OR New Caledonia.tw OR Nicaragua.tw OR Niger.tw OR Nigeria.tw OR Northern Mariana Islands.tw OR Oman.tw OR Muscat.tw OR Pakistan.tw OR Palau.tw OR Palestine.tw OR Panama.tw OR Paraguay.tw OR Peru.tw OR Philippines.tw OR Philipines.tw OR Phillipines.tw OR Phillippines.tw OR Poland.tw OR Portugal.tw OR Puerto Rico.tw OR Romania.tw OR Rumania.tw OR Roumania.tw OR Russia.tw OR Russian.tw OR Rwanda.tw OR Ruanda.tw OR Saint Kitts.tw OR St Kitts.tw OR Nevis.tw OR Saint Lucia.tw OR St Lucia.tw OR Saint Vincent.tw OR St Vincent.tw OR Grenadines.tw OR Samoa.tw OR Samoan Islands.tw OR Navigator Island.tw OR Navigator Islands.tw OR Sao Tome.tw OR Saudi Arabia.tw OR Senegal.tw OR Serbia.tw OR Montenegro.tw OR Seychelles.tw OR Sierra Leone.tw OR Slovenia.tw OR Sri Lanka.tw OR Ceylon.tw OR Solomon Islands.tw OR Somalia.tw OR Sudan.tw OR Suriname.tw OR Surinam.tw OR Swaziland.tw OR Syria.tw OR Tajikistan.tw OR Tadzhikistan.tw OR Tadjikistan.tw OR Tadzhik.tw OR Tanzania.tw OR Thailand.tw OR Togo.tw OR Togolese Republic.tw OR Tonga.tw OR Trinidad.tw OR Tobago.tw OR Tunisia.tw OR Turkey.tw OR Turkmenistan.tw OR Turkmen.tw OR Uganda.tw OR Ukraine.tw OR Uruguay.tw OR USSR.tw OR Soviet Union.tw OR Union of Soviet Socialist Republics.tw OR Uzbekistan.tw OR Uzbek.tw OR Vanuatu.tw OR New Hebrides.tw OR Venezuela.tw OR Vietnam.tw OR Viet Nam.tw OR West Bank.tw OR Yemen.tw OR Yugoslavia.tw OR Zambia.tw OR Zimbabwe.tw OR Rhodesia.tw OR Africa.tw OR Africa, Northern.tw OR Africa South of the Sahara.tw OR Africa, Central.tw OR Africa, Eastern.tw OR Africa, Southern.tw OR Africa, Western.tw OR Asia.tw OR Asia, Central.tw OR Asia, Southeastern.tw OR Asia, Western.tw OR Caribbean Region.tw OR West Indies.tw OR South America.tw OR Latin America.tw OR Central America.tw OR Afghanistan.tw OR Albania.tw OR Algeria.tw OR American Samoa.tw OR Angola.tw OR Antigua.tw OR Barbuda.tw OR Argentina.tw OR Armenia.tw OR Azerbaijan.tw OR Bahrain.tw OR Bangladesh.tw OR Barbados.tw OR Benin.tw OR Byelarus.tw OR Belize.tw OR Bhutan.tw OR Bolivia.tw OR Bosnia-Herzegovina.tw OR Botswana.tw OR Brazil.tw OR Bulgaria.tw OR Burkina Faso.tw OR Burundi.tw OR Cambodia.tw OR Cameroon.tw OR Cape Verde.tw OR Central African Republic.tw OR Chad.tw OR Chile.tw OR China.tw OR Colombia.tw OR Comoros.tw OR Congo.tw OR Costa Rica.tw OR Cote d'Ivoire.tw OR Croatia.tw OR Cuba.tw OR Cyprus.tw OR Czechoslovakia.tw OR Czech Republic.tw OR Slovakia.tw OR Djibouti.tw OR Democratic Republic of the Congo.tw OR Dominica.tw OR Dominican Republic.tw OR East Timor.tw OR Ecuador.tw OR Egypt.tw OR El Salvador.tw OR Eritrea.tw OR Estonia.tw OR Ethiopia.tw OR Fiji.tw OR Gabon.tw OR Gambia.tw OR Georgia.tw OR Ghana.tw OR Greece.tw OR Grenada.tw OR Guatemala.tw OR Guinea.tw OR Guinea-Bissau.tw OR Guam.tw OR Guyana.tw OR Haiti.tw OR Honduras.tw OR Hungary.tw OR India.tw OR Indonesia.tw OR Iran.tw OR Iraq.tw OR Jamaica.tw OR Jordan.tw OR Kazakhstan.tw OR Kenya.tw OR Korea.tw OR Kosovo.tw OR Kyrgyzstan.tw OR Laos.tw OR Latvia.tw OR Lebanon.tw OR Lesotho.tw OR Liberia.tw OR Libya.tw OR Lithuania.tw OR Macedonia.tw OR Madagascar.tw OR Malaysia.tw OR Malawi.tw OR Mali.tw OR Malta.tw OR Mauritania.tw OR Mauritius.tw OR Mexico.tw OR Micronesia.tw OR Middle East.tw OR Moldova.tw OR Mongolia.tw OR Montenegro.tw OR Morocco.tw OR Mozambique.tw OR Myanmar.tw OR Namibia.tw OR Nepal.tw OR Netherlands Antilles.tw OR New Caledonia.tw OR Nicaragua.tw OR Niger.tw OR Nigeria.tw OR Oman.tw OR Pakistan.tw OR Palau.tw OR Panama.tw OR Papua New Guinea.tw OR Paraguay.tw OR Peru.tw OR Philippines.tw OR Poland.tw OR Portugal.tw OR Puerto Rico.tw OR Romania.tw OR Russia.tw OR Rwanda.tw OR Saint Lucia.tw OR Saint Vincent.tw OR Grenadines.tw OR Samoa.tw OR Saudi Arabia.tw OR Senegal.tw OR Serbia.tw OR Montenegro.tw OR Seychelles.tw OR Sierra Leone.tw OR Slovenia.tw OR Sri Lanka.tw OR Somalia.tw OR South Africa.tw OR Sudan.tw OR Suriname.tw OR Swaziland.tw OR Syria.tw OR Tajikistan.tw OR Tanzania.tw OR Thailand.tw OR Togo.tw OR Tonga.tw OR Trinidad.tw OR Tobago.tw OR Tunisia.tw OR Turkey.tw OR Turkmenistan.tw OR Uganda.tw OR Ukraine.tw OR Uruguay.tw OR USSR.tw OR Uzbekistan.tw OR Vanuatu.tw OR Venezuela.tw OR Vietnam.tw OR Yemen.tw OR Yugoslavia.tw OR Zambia.tw OR Zimbabwe.tw OR Burkina Faso.tw OR Upper Volta.tw OR burundi.tw OR Urundi.tw OR cambodia.tw OR Khmer Republic.tw OR Kampuchea.tw OR cameroon.tw OR cameroons.tw OR Cameron.tw OR Camerons.tw OR Cape Verde.tw OR Central African Republic.tw

Web of Science:

- **Date of Download:**
- **N= 1973**

TS=(disaster* OR avalanche* OR blizzard* OR cyclone* OR cyclonic storm* OR drought* OR earthquake* OR famine* OR flood* OR hurricane* OR landslide* OR mudslide* OR tidalwave* OR "tidal wave*" OR tsunami* OR tornado* OR "tropical storm*" OR typhoon* OR wildfire* OR "wild fire*" OR "forest fire*" OR "volcanic eruption*" OR "disease outbreak*" OR epidemic* OR pandemic* OR "hemorrhagic fever*" OR "haemorrhagic fever*" OR ebola OR cholera* OR measles OR starvation OR "nuclear accident*" OR "radiation accident*" OR war* OR conflict* OR "terrorist attack*" OR refugee* OR "asylum seeker*" OR "displaced person*" OR "mass casualt*" OR "emergency responder*" OR "emergency doctor*" OR "emergency clinician*" OR "emergency physician*" OR "emergency personnel" OR "emergency medical personnel" OR "emergency service*" OR "emergency medical service*" OR "emergency medicine" OR "emergency health service*" OR "emergency care" OR "emergency healthcare" OR "emergency treatment*" OR "emergency department*" OR "emergency room*" OR "emergency ward*" OR "emergency unit*" OR "emergency hospital*" OR "emergency clinic*" OR "emergency setting*" OR "emergency staff" OR "emergency response" OR "emergency medical technician*" OR paramedic* OR ambulance* OR "first responder*" OR "rescue work*" OR "relief work*" OR firefighter* OR "fire fighter*" OR "trauma center*" OR "trauma unit*" OR "critical care" OR "critical illness*" OR resuscitation OR shock OR sepsis OR "acute care" OR "acute disease*" OR prehospital OR "pre hospital" OR wound* OR triage OR "pregnancy complication*" OR "obstetric complication*" OR "obstetric emergenc*")
AND
TS=("mobile health" OR mhealth OR "m health" OR "cell* phone*" OR "cell* telephone*" OR "wireless phone*" OR "wireless telephone*" OR "mobile phone*" OR "mobile telephone*" OR "smart phone*" OR smartphone* OR "smart device*" OR "smart watch*" OR smartwatch* OR iPhone* OR "i Phone*" OR android OR blackberry OR "mobile device*" OR "mobile communication*" OR "mobile technolog*" OR "mobile computing" OR "portable electronic device*" OR "mobile electronic device*" OR "handheld device*" OR "hand held device*" OR "handheld computer*" OR "hand held computer*" OR "wireless device*" OR "wireless technolog*" OR "wireless monitor*" OR "wireless sensor*" "personal digital assistant*" OR "tablet computer*" OR "computer tablet*" OR iPad* OR "mobile application*" OR "portable software application*" OR "text messag*" OR texting OR "short messag* service*" OR "SMS" OR "multimedia messag*" OR "multi media messag*" OR "health messag*" OR "wearable electronic device*" OR "wearable computer*" OR "wearable device*" OR "wearable technolog*" OR "wearable sensor*" OR "wearable monitor*" OR telemedicine OR "tele medicine" OR telehealth OR "tele health" OR telehealthcare OR "tele healthcare" OR telemonitoring OR "tele monitoring" OR teleeducation OR "tele education" OR teleconsult* OR "tele consult*" OR telemanagement OR "tele management" OR telediagnosis OR "tele diagnosis" OR teleradiology OR "tele radiology" OR "remote monitor*" OR "remote consult*" OR "remote diagnosis" OR "remote education" OR "videoconferenc*" OR "video conferenc*" OR "clinical decision support")

AND

TS=("developing countr*" OR "developing nation*" OR "developing population*" OR "less developed countr*" OR "less developed nation*" OR "less developed population*" OR "lesser developed countr*" OR "lesser developed nation*" OR "lesser developed population*" OR "lesser developed world" OR "least developed countr*" OR "least developed nation*" OR "least developed population*" OR "least developed world" OR "under developed countr*" OR "under developed nation*" OR "under developed population*" OR "under developed world" OR "underdeveloped countr*" OR "underdeveloped nation*" OR "underdeveloped population*" OR "underdeveloped world" OR "middle income countr*" OR "middle income nation*" OR "middle income population*" OR "low income countr*" OR "low income nation*" OR "low income population" OR "low income population*" OR "lower income countr*" OR "lower income nation*" OR "lower income population*" OR "underserved countr*" OR "underserved nation*" OR "underserved population*" OR "underserved world" OR "under served countr*" OR "under served nation*" OR "under served population*" OR "under served world" OR "deprived countr*" OR "deprived nation*" OR "deprived population*" OR "deprived world" OR "poor countr*" OR "poor nation*" OR "poor population*" OR "poor world" OR "poorer countr*" OR "poorer nation*" OR "poorer population*" OR "poorer world" OR "developing econom*" OR "less developed econom*" OR "lesser developed econom*" OR

"under developed econom*" OR "underdeveloped econom*" OR "middle income econom*" OR "low income econom*" OR "lower income econom*" OR "low gdp" OR "low gnp" OR "low gross domestic" OR "low gross national" OR "lower gdp" OR "lower gnp" OR "lower gross domestic" OR "lower gross national" OR lmic OR lmics OR "third world" OR "lami countr*" OR "transitional countr*" OR Africa OR Asia OR Caribbean OR West Indies OR South America OR Latin America OR Central America OR Afghanistan OR Albania OR Algeria OR Angola OR Antigua OR Barbuda OR Argentina OR Armenia OR Armenian OR Aruba OR Azerbaijan OR Bahrain OR Bangladesh OR Barbados OR Benin OR Byelarus OR Byelorussian OR Belarus OR Belorussian OR Belorussia OR Belize OR Bhutan OR Bolivia OR Bosnia OR Herzegovina OR Hercegovina OR Botswana OR Brasil OR Brazil OR Bulgaria OR Burkina Faso OR Burkina Fasso OR Upper Volta OR Burundi OR Urundi OR Cambodia OR Khmer Republic OR Kampuchea OR Cameroon OR Cameroons OR Cameron OR Camerons OR Cape Verde OR Central African Republic OR Chad OR Chile OR China OR Colombia OR Comoros OR Comoro Islands OR Comores OR Mayotte OR Congo OR Zaire OR Costa Rica OR Cote d'Ivoire OR Ivory Coast OR Croatia OR Cuba OR Cyprus OR Czechoslovakia OR Czech Republic OR Slovakia OR Slovak Republic OR Djibouti OR French Somaliland OR Dominica OR Dominican Republic OR East Timor OR East Timur OR Timor Leste OR Ecuador OR Egypt OR United Arab Republic OR El Salvador OR Eritrea OR Estonia OR Ethiopia OR Fiji OR Gabon OR Gabonese Republic OR Gambia OR Gaza OR Georgia Republic OR Georgian Republic OR Ghana OR Gold Coast OR Greece OR Grenada OR Guatemala OR Guinea OR Guam OR Guiana OR Guyana OR Haiti OR Honduras OR Hungary OR India OR Maldives OR Indonesia OR Iran OR Iraq OR Isle of Man OR Jamaica OR Jordan OR Kazakhstan OR Kazakh OR Kenya OR Kiribati OR Korea OR Kosovo OR Kyrgyzstan OR Kirghizia OR Kyrgyz Republic OR Kirghiz OR Kirgizstan OR "Lao PDR" OR Laos OR Latvia OR Lebanon OR Lesotho OR Basutoland OR Liberia OR Libya OR Lithuania OR Macedonia OR Madagascar OR Malagasy Republic OR Malaysia OR Malaya OR Malay OR Sabah OR Sarawak OR Malawi OR Nyasaland OR Mali OR Malta OR Marshall Islands OR Mauritania OR Mauritius OR Agalega Islands OR Mexico OR Micronesia OR Middle East OR Moldova OR Moldovia OR Moldovian OR Mongolia OR Montenegro OR Morocco OR Ifni OR Mozambique OR Myanmar OR Myanma OR Burma OR Namibia OR Nepal OR Netherlands Antilles OR New Caledonia OR Nicaragua OR Niger OR Nigeria OR Northern Mariana Islands OR Oman OR Muscat OR Pakistan OR Palau OR Palestine OR Panama OR Paraguay OR Peru OR Philippines OR Philipines OR Phillipines OR Phillippines OR Poland OR Portugal OR Puerto Rico OR Rhodesia OR Romania OR Rumania OR Roumania OR Russia OR Russian OR Rwanda OR Ruanda OR Saint Kitts OR St Kitts OR Nevis OR Saint Lucia OR St Lucia OR Saint Vincent OR St Vincent OR Grenadines OR Samoa OR Samoan Islands OR Navigator Island OR Navigator Islands OR Sao Tome OR Saudi Arabia OR Senegal OR Serbia OR Montenegro OR Seychelles OR Sierra Leone OR Slovenia OR Sri Lanka OR Ceylon OR Solomon Islands OR Somalia OR Sudan OR Suriname OR Surinam OR Swaziland OR Syria OR Tajikistan OR Tadzhikistan OR Tadjikistan OR Tadzhik OR Tanzania OR Thailand OR Togo OR Togolese Republic OR Tonga OR Trinidad OR Tobago OR Tunisia OR Turkey OR Turkmenistan OR Turkmen OR Uganda OR Ukraine OR Uruguay OR USSR OR Soviet Union OR Union of Soviet Socialist Republics OR Uzbekistan OR Uzbek OR Vanuatu OR New Hebrides OR Venezuela OR Vietnam OR Viet Nam OR West Bank OR Yemen OR Yugoslavia OR Zambia OR Zimbabwe)

Global Index Medicus:

- **Date of Download: 10.3.19**
- **N=1350 (828 english or spanish)**

Worldwide Disaster
"disaster" OR "disasters" OR "avalanche" OR "avalanches" OR "blizzard" OR "blizzards" OR "cyclone" OR "cyclones" OR "cyclonic storm" OR "cyclonic storms" OR "drought" OR "droughts" OR "earthquake" OR "earthquakes" OR "famine" OR "famines" OR "flood" OR "floods" OR "flooding" OR "hurricane" OR "hurricanes" OR "landslide" OR "landslides" OR "land slide" OR "land slides" OR "mudslide" OR "mudslides" OR "mud slide" OR "mud slides" OR "rockslide" OR "rockslides" OR "rock slide" OR "rock slides" OR "tidalwave" OR "tidalwaves" OR "tidal wave" OR "tidal waves" OR "tsunami" OR "tsunamis" OR "tornado" OR "tornadoes" OR "tropical storm" OR "tropical storms" OR "typhoon" OR "typhoons" OR "wildfire" OR "wildfires" OR "wild fire" OR "wild fires" OR "forest fire" OR "forest fires" OR "volcanic eruption" OR "volcanic eruptions" OR "disease outbreak" OR "disease outbreaks" OR "epidemic" OR "epidemics" OR "pandemic" OR "pandemics" OR "hemorrhagic fever" OR "hemorrhagic fevers" OR "haemorrhagic fever" OR "haemorrhagic fevers" OR "ebola" OR "cholera" OR "measles" OR "starvation" OR "nuclear accident" OR "nuclear accidents" OR "radiation accident" OR "radiation accidents" OR "war" OR "wars" OR "warfare" OR "conflict" OR "conflicts" OR "terrorist attack" OR "terrorist attacks" OR "terrorism" OR "refugee" OR "refugees" OR "asylum seeker" OR "asylum seekers" OR "displaced person" OR "displaced persons" OR "humanitarian" OR "humanitarians" OR "humanitarianism" OR "mass casualty" OR "mass casualties" OR "mass shooting" OR "mass shootings" OR "emergency responder" OR "emergency responders" OR "emergency doctor" OR "emergency doctors" OR "emergency clinician" OR "emergency clinicians" OR "emergency physician" OR "emergency physicians" OR "emergency personnel" OR "emergency medical personnel" OR "emergency service" OR "emergency services" OR "emergency medical service" OR "emergency medical services" OR "emergency medicine" OR "emergency health service" OR "emergency health services" OR "emergency care" OR "emergency healthcare" OR "emergency treatment" OR "emergency treatments" OR "emergency department" OR "emergency departments" OR "emergency room" OR "emergency rooms" OR "emergency ward" OR "emergency wards" OR "emergency unit" OR "emergency units" OR "emergency hospital" OR "emergency hospitals" OR "emergency setting" OR "emergency response" OR "emergency medical technician" OR "emergency medical technicians" OR "paramedic" OR "paramedics" OR "ambulance" OR "ambulances" OR "ER" OR "first responder" OR "first responders" OR "rescue work" OR "rescue worker" OR "rescue workers" OR "relief work" OR "relief worker" OR "relief workers" OR "firefighter" OR "firefighters" OR "fire fighter" OR "fire fighters" OR "trauma center" OR "trauma centers" OR "trauma unit" OR "trauma units" OR "critical care" OR "critical illness" OR "critical illnesses" OR "resuscitation" OR "shock" OR "sepsis" OR "septicemia" OR "septicaemia" OR "acute care" OR "acute disease" OR "acute diseases" OR "prehospital" OR "pre hospital" OR "wound" OR "wounds" OR "triage" OR "pregnancy complication" OR "pregnancy complications" OR "obstetric complication" OR "obstetric complications" OR "obstetric emergency" OR "obstetric emergencies"

Mobile health
"mobile health" OR "mhealth" OR "m health" OR “m-health” OR "cell phone" OR "cell phones" OR "cellular phone" OR "cellular phones" OR "cellular telephone" OR "cellular telephones" OR "wireless phone" OR "wireless phones" OR "wireless telephone" OR "wireless telephones" OR "mobile phone" OR "mobile phones" OR "mobile telephone" OR "mobile telephones" OR "smart phone" OR "smart phones" OR "smartphone" OR "smartphones" OR "smart device" OR "smart devices" OR "smart watch" OR "smart watches" OR "smartwatch" OR "smartwatches" OR "iPhone" OR "iPhones" OR "i Phone" OR "i Phones" OR "android" OR "blackberry" OR "mobile device" OR "mobile devices" OR "mobile communication" OR "mobile communications" OR "mobile technology" OR "mobile technologies" OR "mobile computing" OR "portable electronic device" OR "portable electronic devices" OR "mobile electronic device" OR "mobile electronic devices" OR "handheld device" OR "handheld devices" OR "hand held device" OR "hand held devices" OR "handheld computer" OR "handheld computers" OR "hand held computer" OR "hand held computers" OR "wireless device" OR "wireless devices" OR "wireless technology" OR "wireless technologies" OR "wireless monitor" OR "wireless monitors" OR "wireless monitoring" OR "wireless sensor" OR "wireless sensors" OR "personal digital assistant" OR "personal digital assistants" OR "tablet computer" OR "tablet computers" OR "computer tablet" OR "computer tablets" OR "iPad" OR "iPads" OR "i Pad" OR "i Pads" OR "iPod" OR "iPods" OR "i Pod" OR "i Pods" OR "app" OR "apps" OR "mobile application" OR "mobile applications" OR "portable software application" OR "portable software applications" OR "text message" OR "text messages" OR "text messaging" OR "texting" OR "short message service" OR "short message services" OR "short messaging

service" OR "short messaging services" OR "SMS" OR "health message" OR "health messages" OR "health messaging" OR "wearable electronic device" OR "wearable electronic devices" OR "wearable computer" OR "wearable computers" OR "wearable device" OR "wearable devices" OR "wearable technology" OR "wearable technologies" OR "wearable sensor" OR "wearable sensors" OR "wearable monitor" OR "wearable monitors" OR "telemedicine" OR "tele medicine" OR "telehealth" OR "tele health" OR "telehealthcare" OR "tele healthcare" OR "telemonitoring" OR "tele monitoring" OR "teleeducation" OR "tele education" OR "teleconsult" OR "tele consult" OR "teleconsultation" OR "tele consultation" OR "telemanagement" OR "tele management" OR "telediagnosis" OR "tele diagnosis" OR "teleradiology" OR "tele radiology" OR "remote monitor" OR "remote monitoring" OR "remote consulting" OR "remote consultation" OR "remote consultations" OR "remote diagnosis" OR "remote education" OR "videoconference" OR "video conference" OR "videoconferencing" OR "video conferencing" OR “webcast” OR "clinical decision support"

Search Strategy References:
The search terms were indexed and cross referenced via the following references [2 10 73-78]
